# Effect of the abolition of intersubunit salt bridges on allosteric protein structural dynamics†

**DOI:** 10.1039/d1sc01207j

**Published:** 2021-05-10

**Authors:** Minseo Choi, Jong Goo Kim, Srinivasan Muniyappan, Hanui Kim, Tae Wu Kim, Yunbeom Lee, Sang Jin Lee, Seong Ok Kim, Hyotcherl Ihee

**Affiliations:** Department of Chemistry, Korea Advanced Institute of Science and Technology (KAIST) Daejeon 34141 Republic of Korea hyotcherl.ihee@kaist.ac.kr; KI for the BioCentury, Korea Advanced Institute of Science and Technology (KAIST) Daejeon 34141 Republic of Korea; Center for Nanomaterials and Chemical Reactions, Institute for Basic Science (IBS) Daejeon 34141 Republic of Korea

## Abstract

A salt bridge, one of the representative structural factors established by non-covalent interactions, plays a crucial role in stabilizing the structure and regulating the protein function, but its role in dynamic processes has been elusive. Here, to scrutinize the structural and functional roles of the salt bridge in the process of performing the protein function, we investigated the effects of salt bridges on the allosteric structural transition of homodimeric hemoglobin (HbI) by applying time-resolved X-ray solution scattering (TRXSS) to the K30D mutant, in which the interfacial salt bridges of the wild type (WT) are abolished. The TRXSS data of K30D are consistent with the kinetic model that requires one monomer intermediate in addition to three structurally distinct dimer intermediates (I_1_, I_2_, and I_3_) observed in WT and other mutants. The kinetic and structural analyses show that K30D has an accelerated biphasic transition from I_2_ to I_3_ by more than nine times compared to WT and lacks significant structural changes in the transition from R-like I_2_ to T-like I_3_ observed in WT, unveiling that the loss of the salt bridges interrupts the R–T allosteric transition of HbI. Besides, the correlation between the bimolecular CO recombination rates in K30D, WT, and other mutants reveals that the bimolecular CO recombination is abnormally decelerated in K30D, indicating that the salt bridges also affect the cooperative ligand binding in HbI. These comparisons of the structural dynamics and kinetics of K30D and WT show that the interfacial salt bridges not only assist the physical connection of two subunits but also play a critical role in the global structural signal transduction of one subunit to the other subunit *via* a series of well-organized structural transitions.

## Abstract 2

The structural and functional roles of salt bridges in the allosteric structural transition of homodimeric hemoglobin are investigated by time-resolved X-ray solution scattering.

## Graphical abstract

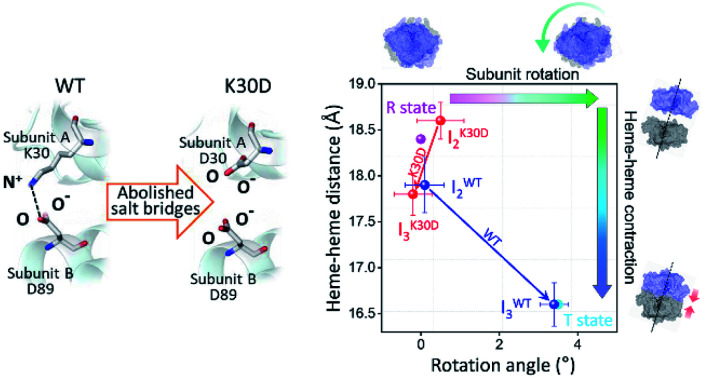

## Introduction

A function of a protein has an intimate connection with its three-dimensional structure. Thus, understanding the interactions inside the protein that affect the structure provides key clues for uncovering the function of a biological system. The structure of a protein is determined by the covalent interactions between amino acid residues to establish the backbone of the protein as well as the non-covalent interactions such as hydrogen bonds, van der Waals interactions, ionic interactions, and hydrophobic interactions that determine how the protein folds. The salt bridge is one of the typical structural factors generated by non-covalent interactions found in many proteins and is formed by a combination of hydrogen bonds and ionic interactions. In a protein, a salt bridge is mainly constructed between a negatively charged carboxylate of aspartic acid or glutamic acid and cationic ammonium of lysine or guanidinium. Many studies have been conducted to unveil the structural and functional roles of salt bridges in proteins. The salt bridge in the structural aspect mainly plays a role in stabilizing entropically unfavorable conformations, enhancing the stabilities of secondary, tertiary, and quaternary structures, and having resistance to protein aggregation.^1,2^ Since the salt bridge contributes to stabilizing the protein structure, the protein activity is inevitably regulated by the salt bridge. Many cases have been reported where the proteins cannot perform their original functions when the salt bridges are abolished.^2–4^ Although much research has been conducted on salt bridges due to their importance in determining the structures and functions, most studies have been conducted only on static equilibrium states. In reality, however, performing a protein function is not a static process in which the protein stays in a specific structure but a dynamic process in which the structure changes over time. Therefore, it is important to identify how the salt bridge affects the structural dynamics of a protein, for example, the structures of reaction intermediates and the associated reaction rates.

In this regard, here we investigated how the structural dynamics of a protein are altered in the presence or absence of salt bridges to improve the understanding of the structural and functional role of the salt bridges in the course of the dynamic biological process. Specifically, we investigated the role of the salt bridges during the allosteric structural transition of homodimeric hemoglobin (HbI) from *Scapharca inaequivalvis*. Allosteric regulation is one of the essential mechanisms regulating the function of proteins, in which the structural change at one site leads to significant feedback at a remote site of the same macromolecular entity. A well-known example is human tetrameric hemoglobin (Hb), where the binding of dioxygen or carbon monoxide is known to benefit from allosteric regulation.^5–12^ While the tetrameric structure of Hb makes detailed experimental studies difficult,^13^ HbI offers a greatly simplified model system, thanks to its homodimeric structure. Despite being only a dimer, the characteristics of cooperative ligand binding and allosteric structural transitions are clearly expressed between the liganded R state with high ligand affinity and the unliganded T state with low ligand affinity.^14–26^

**Fig. 1:**
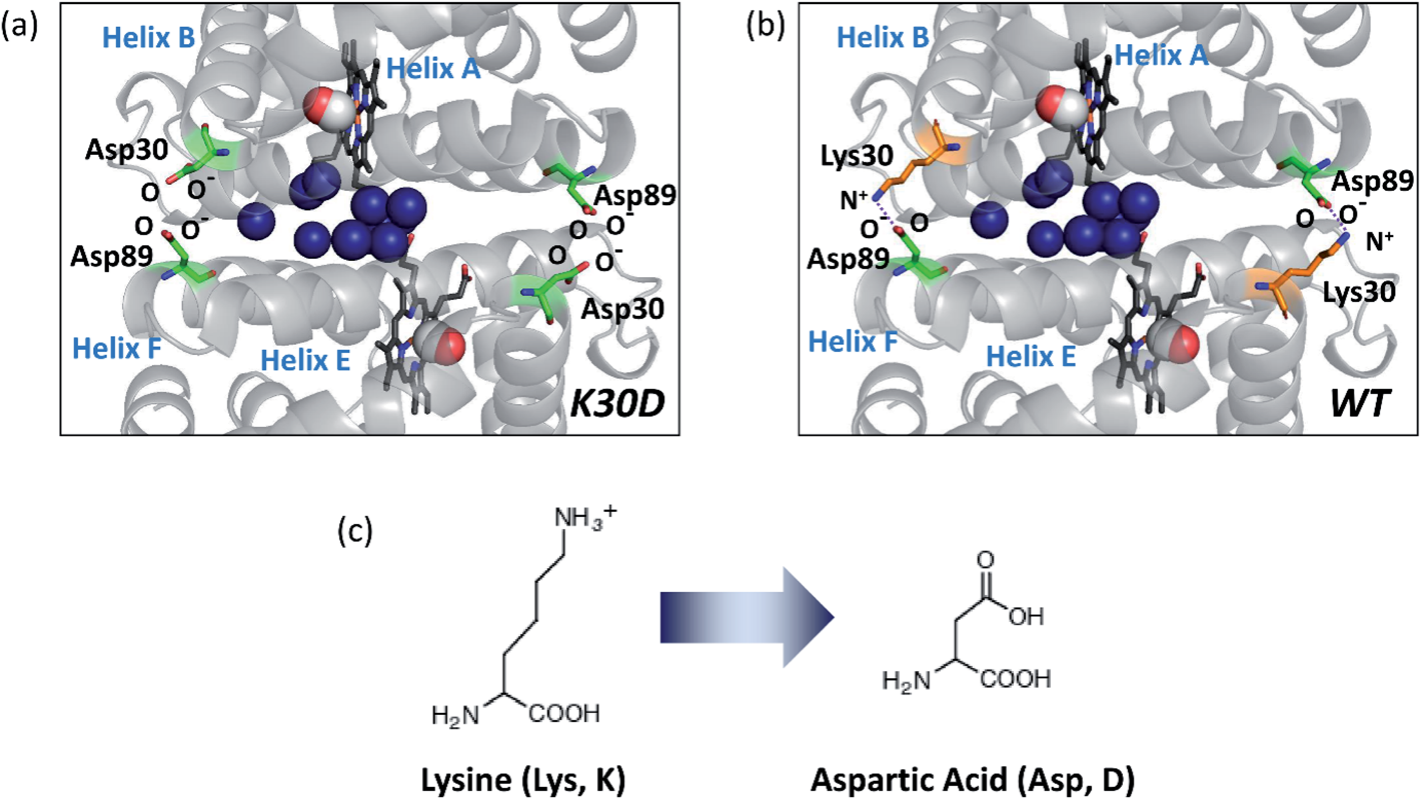
Subunit interface of HbI(CO)_2_. (a) Structure of K30D HbI(CO)_2_. (b) The crystal structure of WT HbI(CO)_2_ (PDB ID: 3SDH). The structure of K30D is based on the crystal structure of WT. In K30D, the Lys30 residue (shown in orange) in each subunit of WT is replaced by Asp30 (shown in green) as shown in (c). In (a) and (b), two carbon monoxide molecules are shown with connected red and white spheres. Eleven interfacial water molecules (shown in dark blue) are well organized and connected to two subunits and hemes. In WT, the network of the subunit interface is stabilized by symmetrical salt bridges between Lys30 and Asp89. In contrast, the interface is destabilized in K30D.

HbI has two symmetrically linked salt bridges between Lys30 and Asp89 at the subunit interface. The salt bridges are one of the essential factors composing the intersubunit network and stabilizing the dimeric assembly with two subunits connected. By two symmetrically linked intersubunit salt bridges between Lys30 in one subunit and Asp89 in the other subunit, one subunit may communicate to the other subunit. The K30D mutation, where the Lys30 residues of the two subunits consisting of the salt bridges of the wild type (WT) are replaced with Asp30 residues, abolishes the only salt bridges at the subunit interface by introducing charge repulsion between Asp30 and Asp89 (Fig. 1) so that this mutant is a relevant system for identifying the effect of the salt bridges on the structural dynamics of HbI.

A study using spectroscopic characterization on K30D reported the dimerization constants of unliganded and liganded derivatives, the oxygen equilibrium measurements, and the kinetics of oxygen release.^27^ It was reported that the cleavage of the salt bridges destabilizes the intersubunit network and dimeric assembly of HbI, resulting in a change in the mechanism of cooperativity.^27^ According to the reported dimerization constant,^27^ where WT mostly exists as the dimeric form in nature, the fraction of the monomer is not negligible in K30D due to the weak interaction at the interface. The local structural changes near the heme pocket and the kinetics upon oxygen release in K30D were investigated by spectroscopic studies and equilibrium oxygen-binding experiments.^27^ Still, more detailed kinetic and structural information, such as lifetimes and tertiary and quaternary structures of reaction intermediates, is unclear. In this regard, time-resolved X-ray solution scattering (TRXSS)^28–33^ can provide complementary dynamic information on the allosteric structural transition of K30D in solution for unveiling the effect of the salt bridges in HbI on the structural dynamics. TRXSS, also known as time-resolved X-ray liquidography (TRXL), is a useful technique that offers complementary advantages over conventional methods, as it allows for easy observation of the structural dynamics of proteins in physiological environments.

In this work, to elucidate how the interfacial salt bridges play a role in the allosteric structural transition in HbI, we performed the TRXSS experiment on K30D in solution and extracted the structures of the intermediates and determined kinetic parameters based on the modified kinetic model that considered the monomer fraction. In particular, we obtained the detailed structural parameters of the intermediates of K30D HbI, such as root-mean-square deviations (RMSDs), displacement plots, subunit rotation angles, heme–heme distances, and distances between Cα atoms of the key residues involved in the salt bridges in WT HbI, and quantitatively compared these structural parameters with those of WT HbI. We found that all the three dimer intermediates (I_1_, I_2_, and I_3_) of K30D have R-like structures, unlike WT involving a transition from R-like I_1_ and I_2_ to T-like I_3_. These observations indicate that the dramatic structural change associated with the R–T transition is remarkably suppressed by losing the salt bridges in K30D. Considering that the bimolecular CO recombination is decelerated in K30D although I_3_ of K30D has a structure more similar to the ground state compared to that of WT, the loss of the salt bridges may diminish the ligand-binding affinity and eventually make the CO recombination process slow.

## Experimental

### Sample preparation

The K30D mutation was introduced into the native recombinant HbI plasmid using the EZchange™ site-directed mutagenesis kit (Enzynomics) with the following oligonucleotide encoding K30D mutation: 5′-GGTTCGGACAAAGATGGTAACGG-3′ and 5′-CCGTTACCATCTTTGTCCGAACC (Genotech). The K30D mutant (K30D) was over-expressed in *E. coli* and purified according to the method^34^ described for WT. Carbonmonoxy derivatives of K30D solution for the photodissociation reaction were prepared as follows. A 2–4 mM deoxy K30D solution in 100 mM phosphate buffer (pH 7) was prepared in a rubber-topped air-tight vial. The protein concentration was determined from the absorbance at 578 nm using the absorption coefficient of heme-oxygenated derivatives (14.3 mM^−1^ cm^−1^). The deoxy K30D was reduced by adding 10 μL of 1 M sodium dithionite solution under a nitrogen atmosphere. The reduced samples were exposed to CO gas for 30 minutes to convert deoxy to CO-bound K30D. The sample solution was prepared just before the X-ray solution scattering measurement. An aliquot of the resulting CO-bound K30D solution was transferred into a quartz capillary with a 1 mm diameter (Hampton Research) and immediately sealed with epoxy to minimize gas exchange while CO gas was purged continuously into the capillary.

### Data acquisition

The TRXSS experiment of K30D was conducted at the BioCARS beamline 14IDB at the Advanced Photon Source using the experimental setup of a typical pump-probe method. Circularly polarized laser pulses generated from a picosecond laser system were sent to an optical parametric amplifier (TOPAS) to generate output pulses with a center wavelength of 532 nm and a temporal duration of ∼35 ps. The laser light was focused on 0.15 × 0.60 mm^2^ at the sample position and finally had an energy density of 1 mJ mm^−2^. The X-ray pulse peaked at 12 keV with a long wavelength tail of ∼4% and a photon flux of ∼10^9^ photons/pulse. The size of the X-ray beam was adjusted to be 0.09 × 0.07 mm^2^ at the sample position, which was smaller than the laser spot size, to minimize the timing jitter caused by the spatial error between the laser and X-ray pulses. After a laser pulse passed through the sample to induce the reaction, an X-ray pulse arrived with a time delay, Δ*t*, and time-resolved X-ray scattering data were measured at time delays in the range from 100 ps to 10 ms. The helium cone was utilized to reduce the air scattering by X-ray. Two-dimensional (2D) scattering patterns were collected using an area detector (MarCCD) located at a distance of 185 mm from the sample position. The capillary containing the protein sample was moved along the axis perpendicular to the pulse train of X-ray and the laser and was set to perform repetitive motion in the forward and backward directions to provide a fresh sample for each X-ray and laser pair. The obtained 2D X-ray scattering patterns have centrosymmetry due to the random orientation of the molecules in the solution phase, so that one-dimensional (1D) X-ray scattering curves can be obtained by azimuthally integrating the patterns as a function of the magnitude of the momentum transfer vector, *q* = (4π/*λ*)sin(2*θ*/2) where *λ* is the X-ray wavelength and 2*θ* is the scattering angle. The 1D X-ray scattering curves, *S*(*q*, Δ*t*), contain information about the X-ray scattering occurring from the solvent pairs, solute pairs, and solvent–solute pairs at the corresponding time delay. The X-ray scattering signal from the solvent molecule was much larger than the signal of our interest resulting from the reaction of the solute molecules. The scattering curve at the negative time delay, −5 μs, containing the signal from the unreacted sample solution was subtracted from the scattering curve for each time delay, and the scattering signal generated by the bulk solvent was removed by this process. As a result, we obtained the time-resolved difference X-ray solution scattering curves, Δ*S*(*q*, Δ*t*). The time delays used in the experiments are as follows:

−5 μs, 100 ps, 178 ps, 316 ps, 562 ps, 1 ns, 1.78 ns, 3.16 ns, 5.62 ns, 10 ns, 17.8 ns, 31.6 ns, 56.2 ns, 100 ns, 178 ns, 316 ns, 562 ns, 1 μs, 1.78 μs, 3.16 μs, 5.62 μs, 10 μs, 17.8 μs, 31.6 μs, 56.2 μs, 100 μs, 178 μs, 316 μs, 562 μs, 1 ms, 1.78 ms, 3.16 ms, 5.62 ms, and 10 ms.

The measured difference X-ray scattering curves contained the signal from the thermal heating of the solvent. For the data analysis, we removed the thermal heating contribution using a well-established method.^35–38^ During the experiment, the temperature was maintained at 25 °C by a stream of cold nitrogen gas (Oxford Cryostream).

## Results and discussion

### Kinetic analysis of time-resolved difference solution scattering curves

**Fig. 2:**
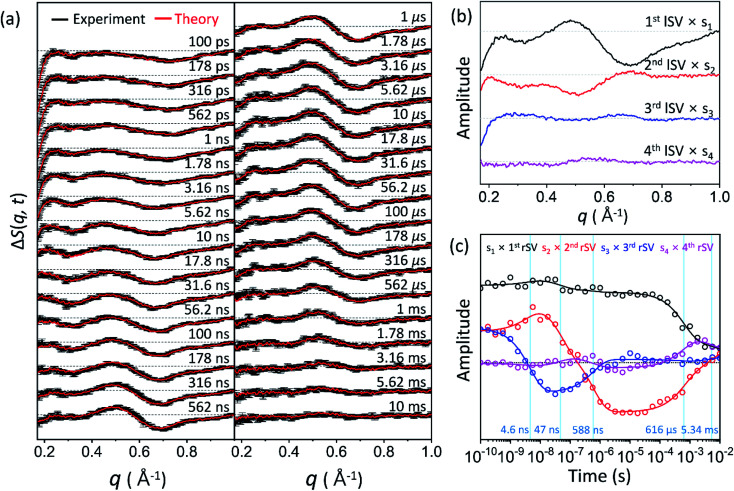
(a) Time-resolved X-ray solution scattering curves, Δ*S*(*q*, *t*), at time delays from 100 ps to 10 ms were obtained for the K30D mutant of HbI in solution. Experimental curves with standard deviation (shown in black) are compared with theoretical curves (shown in red) generated from a linear combination of left singular vectors (lSVs) extracted from the kinetic analysis. (b) The first four lSVs were multiplied by singular values (Fig. S9†) from singular value decomposition (SVD) analysis. (c) The first four right singular vectors (rSVs) were multiplied by singular values and fitted by the sum of exponential functions sharing five time constants of 4.6 ns, 47 ns, 588 ns, 616 μs, and 5.34 ms.

Time-resolved difference X-ray solution scattering curves, Δ*S*(*q*, *t*), obtained for time delays (*t*) from 100 ps to 10 ms are shown in Fig. 2a. The data exhibit oscillatory features along the *q*-axis due to protein structural changes. To extract kinetic information of intermediates and their structures from Δ*S*(*q*, *t*), we followed the well-established procedure^35–38^ which had been applied in previous TRXSS studies on WT and various mutants of HbI, consisting of kinetic analysis using singular value decomposition (SVD) and principal component analysis (PCA). As detailed in the ESI,† SVD and the global fit of left singular vectors (lSVs) show that the kinetics involves four structurally distinct intermediates and five time constants of 4.6 (±0.7) ns, 47 (±13) ns, 588 (±81) ns, 616 (±109) μs, and 5.34 (±5.22) ms. The kinetics of all the HbI proteins so far studied with TRXSS, including WT, F97Y,^37^ T72V,^36^ and I114F^35^ can be explained with the same kinetic framework with only minor variations. This framework has three intermediates, a transition from the first intermediate to the second intermediate (I_1_ → I_2_), a biphasic transition from the second intermediate to the third intermediate (I_2_ → I_3_), and bimolecular recombination to recover CO-bound HbI(CO)_2_. Compared to the WT data, which exhibit three intermediates and seven time constants, K30D has one additional intermediate and a smaller number of kinetic components, requiring a more significant alteration to the known the kinetic framework.

**Fig. 3:**
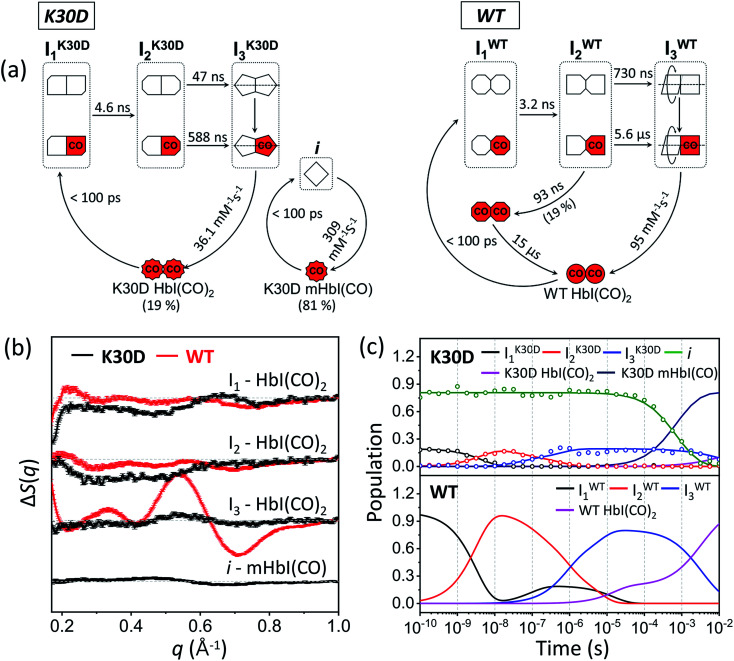
(a) Kinetic models for K30D (left) and WT (right). The red (with “CO”) and white symbols represent ligated and photolyzed subunits, respectively. The subunits of each intermediate are represented in different shapes to describe the change in the tertiary structure with the progress of transition from I_1_ to I_2_. To indicate quaternary structure change for the transition from I_2_ to I_3_, one subunit of I_3_ is described as rotation with respect to the other. To indicate the photolyzed monomer, i, the diamond-shaped symbols are used. For WT, two red octagons represent a ligated form of I_1_ formed by geminate recombination of CO with I_2_ and is structurally indistinguishable from the photolyzed forms of I_1_. (b) Species-associated difference scattering curves (SADSs) of the intermediates of K30D (black) and WT (red) HbI. (c) Population changes of the intermediates and initial HbI(CO)_2_ of K30D (upper graph) and WT (lower graph) extracted from the kinetic analysis. The open circles represent the optimized populations obtained by fitting the experimental curve at each time point with a linear combination of SADSs for the four intermediates.

To establish the kinetic framework (Fig. 3a), we used two effective methods that can greatly facilitate narrowing down the kinetic models compatible with the experimental data: (i) the number of species from the SVD results on various reduced time ranges^39^ in addition to the whole data and (ii) SVD-aided pseudo PCA analysis (SAPPA).^39^ As detailed in the ESI,† such analyses reveal that the first time constant of 4.6 ns can be attributed to the transition from I_1_ to I_2_, and the two time constants of 47 ns and 588 ns need to be assigned to the biphasic transition from I_2_ to I_3_. As a result, among five time constants, two (616 μs and 5.34 ms) remain to be assigned, and the identity of one intermediate among four remains to be determined.

We considered the possibility that this remaining intermediate is either a monomer or a dimer and built the kinetic models for both scenarios (Fig. S2a and b,† respectively). The calculated curves from both kinetic models (Fig. S2c and d,† respectively) gave equally satisfactory agreements with the experimental data, meaning that the fit quality alone cannot discriminate whether the fourth species is a dimer or a monomer. Nevertheless, we favor the possibility of a “monomer” intermediate for the following reasons. (i) K30D has a weakened network of interactions between Asp30 and Asp89 located at the subunit interface. K30D has a dimerization constant of 1.2 × 10^3^ M^−1^ at 10 °C under the equivalent conditions,^27^ and the ratio of the monomer is 41% in an aqueous solution. (ii) The monomeric unit of K30D can be regarded as a monomeric heme protein, whose photoinduced structural changes were observed in many time-resolved studies,^28,40–55^ and thus it is plausible that K30D monomer undergoes the structural change induced by photoexcitation. (iii) The simulated difference scattering intensity of the monomer shows a magnitude that cannot be neglected compared to that of the dimer (as detailed in the ESI and Fig. S3†). This result demonstrates that if the dimer intermediates can be distinguished in the TRXSS signals, the monomer intermediate should also be distinguished. Therefore, we considered the possibility of the monomer intermediate in the kinetic model. To avoid any confusion, from now on, intermediates refer to the dimer intermediates and *i* refers to the monomer intermediate. A more detailed explanation for the process of choosing the best kinetic model is described in the ESI.†

Finally, species-associated difference scattering curves (SADSs) and time-dependent concentrations of K30D are compared with those of WT in Fig. 3. To distinguish the intermediates of WT and K30D, we labeled I_1_, I_2_, and I_3_ of WT as I^WT^_1_, I^WT^_2_, and I^WT^_3_, respectively, and likewise labeled the intermediates of K30D as I^K30D^_1_, I^K30D^_2_, and I^K30D^_3_, respectively. For K30D, we labeled the monomeric form of the initial state as K30D mHbI(CO) and the dimeric form of the initial state as K30D HbI(CO)_2_. According to the determined kinetic model, K30D HbI(CO)_2_ is converted to the earliest intermediate, I^K30D^_1_, within the experimental time resolution (<100 ps), and I^K30D^_1_ transforms to I^K30D^_2_ with a time constant of 4.6 ns, which is slightly larger than that of WT (3.2 ns), indicating that the absence of salt bridges affects the earliest intermediates although the effect is not severe. K30D shows the biphasic I^K30D^_2_-to-I^K30D^_3_ transition, as in WT and other mutants, with time constants of 47 ns and 588 ns, which are smaller than those of WT (730 ns and 5.6 μs) by factors of 15.5 and 9.5, respectively, indicating acceleration with respect to WT. According to TRXSS studies on WT and mutants,^35–38^ this I^WT^_2_-to-I^WT^_3_ transition accounts for the quaternary structural change where both the rotation angle and the heme–heme distance significantly changes. Thus, the accelerated rates mean that either (i) these structural changes occur more rapidly or (ii) different structural changes are involved in K30D. This result suggests that the salt bridges between the two subunits may be involved in controlling the quaternary structural change. Then, I^K30D^_3_ returns to K30D HbI(CO)_2_ with a bimolecular rate constant of 36 (±3) mM^−1^ s^−1^, which is nearly three times smaller than that of WT (95 mM^−1^ s^−1^). For the monomeric form of K30D, initial K30D mHbI(CO) is converted to *i*, within the experimental time resolution (<100 ps), and returns to K30D mHbI(CO) with a bimolecular rate constant of 309 (±9) mM^−1^ s^−1^, which is more than eight times larger than that of I^K30D^_3_. The fit result shows 81 (±0.4) % for the fraction of monomers. The reported value at 10 °C is 41%.^27^ The fact that the TRXSS experiment was carried out at 25 °C can rationalize the higher monomer content. The estimated dimerization constant based on the monomer fractions at 25 °C is ∼77 (±1) M^−1^. We note that the relative portion of dimers in terms of the concentration that contributes to the difference scattering signal is ∼20% (and that of monomers is ∼80%), but this does not mean that the relative contribution to the difference scattering signal is 20%. One dimer contributes more strongly to the scattering signal than one monomer because a dimer is two times heavier than a monomer and a dimer generally has a larger structural change than a monomer. The relative contributions of dimers to the difference signal range from 25% to 88% depending on time, as shown in Fig. S12.†

We note that the intermediates of K30D have two substrates of the fully photolyzed and partially photolyzed forms, which are structurally indistinguishable from each other. The same was revealed from previous laser power dependency studies on WT and other mutants,^35–38^ which provided direct evidence that the partially photolyzed dimer subunit undergoes the same structural evolution as the fully photolyzed subunit, directly demonstrating the allosteric regulation of HbI.

### Structural analysis of intermediates

#### Structure refinement aided by Monte-Carlo simulations

The information about the 3D structures of the intermediates can be obtained *via* structural analysis using the SADSs of the intermediates. As shown in Fig. 3, the SADSs of WT and K30D are very different from each other for all intermediates, so it can be seen that all intermediates of K30D are structurally different from WT intermediates. In other words, the mutation from Lys30 to Asp30 changes the structure of all the intermediates. In the case of WT, F97Y and T72V mutants, the SADSs of I_1_ and I_2_ are not much different from each other, suggesting that the structure of I_1_ and I_2_ are not affected by the F97Y or T72V mutation. The SADSs of I_3_ for F97Y and T72V, however, are significantly different compared to WT, indicating that the T72V and F97Y mutations alter only the structure of I_3_. To extract key structural parameters of each intermediate structure, we performed structure refinement applied with a rigid-body modeling approach using crystallographic structures as template structures, which is established in our previous study on WT.^35–38^ In the case of K30D, since its crystallographic structures were not reported, we made the template structures for the structural analysis by modifying the crystallographic structures of WT (see the ESI† for details). From the structure refinement, the refined candidate structures for the intermediates were obtained, and their theoretical scattering curves show excellent agreement with the experimental SADSs (Fig. S4†).

#### Structural analysis of the dimer intermediates of the K30D mutant: RMSDs

**Fig. 4:**
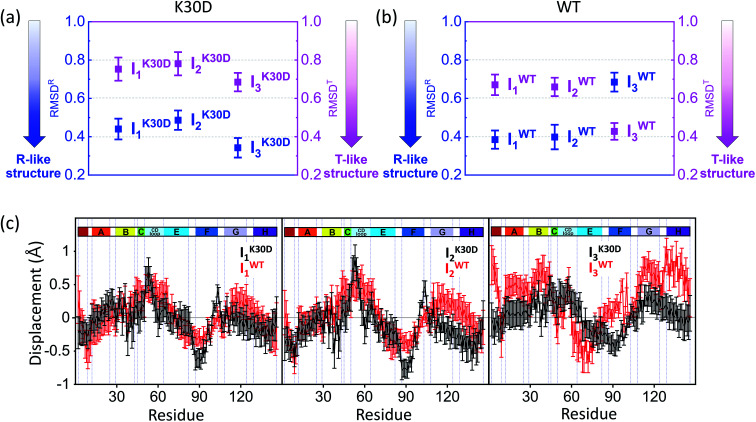
Root-mean-square deviations (RMSDs) were calculated from the candidate structures of the intermediates of (a) K30D and (b) WT. As described in the main text, RMSD^R^ (blue) and RMSD^T^ (magenta) were calculated with respect to the reference structures (for WT, the crystallographic structures and for K30D, modified structures based on these; see the ESI† for details) of the liganded state (R state) and the unliganded state (T state), respectively. The smaller RMSD indicates the structure closer to the corresponding reference structure. The plots display that all intermediates of K30D including I_3_ have the R-like structure rather than the T-like structure. In contrast, in WT, only I_1_ and I_2_ have the R-like structure, while I_3_ has the T-like structure. (c) Averaged displacement plots for K30D (black line) and WT (red line) and for I_1_ (left), I_2_ (middle), and I_3_ (right) were calculated with respect to K30D HbI(CO)_2_ (mutated from 3SDH) and HbI(CO)_2_ (3SDH), respectively. Error bars in (a)–(c) represent standard deviation values among various candidate structures of each intermediate. K30D and WT show similar displacement patterns for I_1_ and I_2_ whereas they show relatively more dissimilar patterns for I_3_.

The key structural parameters, such as the RMSD, displacement of the residues, heme–heme distance, rotation angle, and for the intermediates. To compare the overall structural differences between the intermediates of WT and K30D HbI, we calculated RMSD values in the positions of the Cα atoms with respect to the reference structures (for WT, crystallographic structures of HbI(CO)_2_ (PDB ID: 3SDH) and unliganded HbI (PDB ID: 4SDH) and for K30D, modified structures based on these; see the ESI^†^ for details), termed RMSD^R^ and RMSD^T^, respectively, as shown in Fig. 4a and b. Comparing RMSD^R^ and RMSD^T^ values of the intermediate allows for quantifying the degree of resemblance of the R-like structure or the T-like structure. In previous studies, it was found that I^WT^_1_ and I^WT^_2_ have R-like structures, whereas I^WT^_3_ has a T-like structure.^35–38^ Actually, I^WT^_1_ (RMSD^R^: 0.4 ± 0.05 Å and RMSD^T^: 0.7 ± 0.05 Å) and I^WT^_2_ (RMSD^R^: 0.4 ± 0.06 Å and RMSD^T^: 0.7 ± 0.05 Å) have larger RMSD^T^ than RMSD^R^, whereas I^WT^_3_ (RMSD^R^: 0.7 ± 0.05 Å and RMSD^T^: 0.4 ± 0.04 Å) has a larger RMSD^R^ compared to RMSD^T^, confirming that I^WT^_1_ and I^WT^_2_ have R-like structures and I^WT^_3_ has a T-like structure. For K30D, I^K30D^_1_ (RMSD^R^: 0.4 ± 0.05 Å and RMSD^T^: 0.8 ± 0.06 Å) and I^K30D^_2_ (RMSD^R^: 0.5 ± 0.05 Å and RMSD^T^: 0.8 ± 0.06 Å) exhibit larger RMSD^T^ than RMSD^R^, as in WT, indicating that I^K30D^_1_ and I^K30D^_2_ have R-like structures. However, the opposite trend was observed between I^WT^_3_ and I^K30D^_3_. I^K30D^_3_ (RMSD^R^: 0.3 ± 0.05 Å and RMSD^T^: 0.7 ± 0.05 Å) has a larger RMSD^T^ than RMSD^R^, indicating that I^K30D^_3_ has an R-like structure, unlike the T-like I^WT^_3_. The fact that all the intermediates of K30D have R-like structures suggests that abolishing the salt bridges in K30D restricts the efficient R–T transition in HbI.

#### Tertiary structural changes: displacement plots

To identify the detailed tertiary structural changes, we calculated the displacement plots of the three intermediates, as shown in Fig. 4c. The displacement of the residue is defined as the difference in distance between the Cα and the iron atom of heme in the same subunit, with respect to the reference structure. The crystallographic structures of the liganded states of WT and K30D were used as the reference structures for calculating the displacement plots of WT and K30D intermediates, respectively. In I_1_ and I_2_, the magnitudes and directions of the displacements in K30D are similar to those in WT. In the case of I_3_, however, the magnitudes and directions of the displacements in K30D are different from those in WT. The displacement of I^K30D^_3_ is similar to those of I^WT^_1_ and I^WT^_2_ than that of I^WT^_3_, meaning that the tertiary structure of I^K30D^_3_ has an R-like structure in contrast to I^WT^_3_ having a T-like structure. These trends are also consistent with the results obtained from the comparison of the RMSD values. We note that the displacements and RMSD values of I^K30D^_3_ are between those of I^K30D^_2_ and K30D HbI(CO)_2_, suggesting that I^K30D^_3_ has a tertiary structure located between I^K30D^_2_ and K30D HbI(CO)_2_ structures. This consideration indicates that, unlike WT, the tertiary structure of I^K30D^_3_ returns to that of K30D HbI(CO)_2_ without undergoing the R–T transition occurring in the I^WT^_2_-to-I^WT^_3_ transition. I^WT^_3_ undergoes a rearrangement process in which the amplitude of displacement changes dramatically, whereas this process is suppressed in I^K30D^_3_. These results confirm that the internal helices as well as the interface are affected by the absence of interfacial salt bridges.

#### Quaternary structural changes: heme–heme distances and subunit rotation angles

**Fig. 5:**
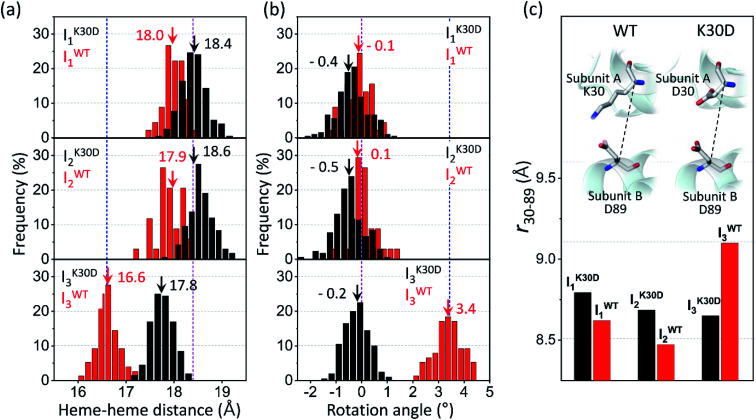
Occurrence distributions of (a) the heme–heme distance and (b) the rotation angle for the intermediates of K30D (black) and WT (red). The average values of K30D and WT are indicated with downward black and red arrows, respectively, for each intermediate. For comparison, the heme–heme distances and the rotation angles of the unliganded form (PDB ID: 4SDH) and the liganded form (PDB ID: 3SDH) of WT are indicated by vertical blue and magenta dotted lines, respectively. For all three intermediates, K30D undergoes smaller changes in the heme–heme distance compared to WT. In addition, the degree of rotation angles also shows that the I_3_ intermediate of K30D undergoes much less rotation than that of WT. (c) The average distance between Cα atoms in 30th and 89th residues, *r*_30–89_, for K30D (black) and WT (red). The 30th and 89th residues are involved in the interfacial salt bridges in WT.

To inspect the detailed quaternary structural changes, we calculated the heme–heme distance and subunit rotation angle of the three intermediates, as shown in Fig. 5a and b. They are the key structural parameters for quantifying the quaternary structural transition since the cooperative ligand binding of HbI is modulated by the hydrogen-bonding network between two hemes and the subunit rotation. In WT, the transition from HbI(CO)_2_ (heme–heme distance = 18.4 Å and subunit rotation angle = 0°) to I^WT^_1_ (18.0 ± 0.2 Å and −0.1 ± 0.5°) and I^WT^_2_ (17.9 ± 0.3 Å and 0.1 ± 0.5°) involves very small changes in the heme–heme distances and the subunit rotation angles, indicating that I^WT^_1_ and I^WT^_2_ have the R-like structures. The major quaternary structural changes occur in the I^WT^_2_-to-I^WT^_3_ transition (16.6 ± 0.2 Å and 3.5 ± 0.6°), indicating that I^WT^_3_ has a T-like structure, unlike I^WT^_1_ and I^WT^_2_. In K30D, the heme–heme distances and subunit rotation angles of both I^K30D^_1_ (18.4 ± 0.3 Å and −0.4 ± 0.6°) and I^K30D^_2_ (18.6 ± 0.2 Å and 0.5 ± 0.6°) are similar to those of the initial HbI(CO)_2_ as in I^WT^_1_ and I^WT^_2_. Besides, the heme–heme distance of I^K30D^_3_ (17.8 ± 0.2 Å) is closer to that of HbI(CO)_2_ than that of unliganded HbI (16.6 Å), unlike I^WT^_3_, which has the T-like structure. The subunit rotation angle of I^K30D^_3_ (−0.2 ± 0.5°), however, is even closer to that of the initial HbI(CO)_2_, whereas the subunit rotation angle of I^WT^_3_ is close to that of unliganded WT HbI. Therefore, the subunit rotation angle of I^K30D^_3_ is much smaller than that of T-like I^WT^_3_. The suppression of the structural rearrangement observed in I^WT^_3_, such as the contraction of the heme–heme distance and the intersubunit rotation, is consistent with the results obtained by considering the RMSD values and displacement plots. The suppressed quaternary structural changes in K30D may be caused by the weakened inter-subunit interaction of K30D, suggesting that the communication by salt bridges is an essential element for normal allosteric transition in HbI. This restricted R–T transition can account for the acceleration of the I^K30D^_2_-to-I^K30D^_3_ transition observed in K30D since the transition in K30D does not require significant quaternary structural changes in contrast to WT. Therefore, it takes less time for the I_2_-to-I_3_ transition to occur in K30D than in WT.

#### Distance between Cα atoms of key residues involved in the salt bridges of WT

We also inspected and averaged the distance between Cα atoms of the 30th residue at one subunit interface and 89th residue at the other subunit interface (denoted *r*_30–89_). The 30th and 89th residues originally form the interfacial salt bridges in WT, but the salt bridges are abolished in K30D. We tried to identify how the movements of the helices connected by the salt bridges in WT are affected by the cleavage of the salt bridges in K30D. For this purpose, we compared the *r*_30–89_ of all the intermediates of WT and K30D, as shown in Fig. 5c. I^K30D^_1_ has a longer *r*_30–89_ (8.8 ± 0.2 Å) than I^WT^_1_ (8.6 ± 0.3 Å), and also I^K30D^_2_ has a longer *r*_30–89_ (8.7 ± 0.2 Å) than I^WT^_2_ (8.5 ± 0.3 Å). The longer *r*_30–89_ distances in K30D than in WT indicate that the two residues are connected less tightly in K30D. The *r*_30–89_ values in both K30D and WT become smaller in the I_1_-to-I_2_ transition, and the contraction magnitudes (∼0.1 Å) are maintained similarly in both K30D and WT. The *r*_30–89_ of I^K30D^_3_ (8.7 ± 0.2 Å) is smaller than that of I^K30D^_2_, whereas I^WT^_3_ (9.1 ± 0.2 Å) has an increased *r*_30–89_ compared to I^WT^_2_. This trend is in good agreement with that the I_2_^WT^-to-I^WT^_3_ transition contains a large intersubunit rotation compared to the I^K30D^_2_-to-I^K30D^_3_ transition. These results indicate that the *r*_30–89_ is closely linked to the interfacial salt bridges and the quaternary structural changes such as the subunit rotation angle and suggest that the interfacial salt bridges have a critical role in maintaining the proper distances between helices, and in having the sufficient magnitude of subunit rotation angle in the R–T transition.

#### Correlation between the bimolecular CO recombination rate and quaternary structure of I_3_

**Fig. 6:**
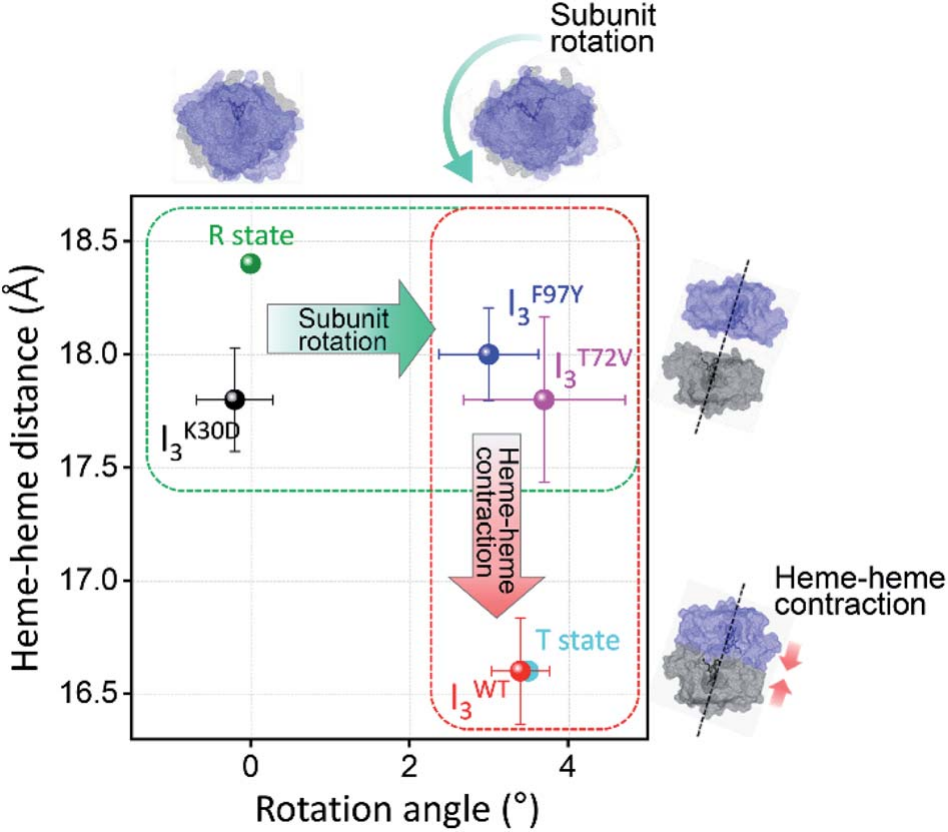
Heme–heme distances of the I_3_ intermediates of WT and various mutants including K30D are plotted as a function of the subunit rotation angle. For comparison, those of R and T states of WT, that is the crystallographic structures of HbI(CO)_2_ and unliganded HbI, respectively, are also shown as dots in green and cyan. The dots with errors in black, red, blue, magenta correspond to averaged candidate structures of I^K30D^_3_, I^WT^_3_, I^F97Y^_3_, and I^T72V^_3_, respectively. Error bars represent standard deviation values among various candidate structures of each intermediate.

To infer the functional role of the salt bridges in cooperative ligand binding in HbI, we examined the results of the kinetic and structural analyses. Noticeably, the bimolecular CO recombination rate of I^K30D^_3_ (36.1 mM^−1^ s^−2^) is slower than that of I^WT^_3_ (95 mM^−1^ s^−2^), although I^K30D^_3_ has an R-like structure. Intuitively, since I^K30D^_3_ has a structure highly are similar to the ground state, the recovery process of I^K30D^_3_ is expected to be faster than that of I^WT^_3_. In fact, in the case of F97Y and T72V mutants, whose structural dynamics were investigated using the TRXSS technique, the more the quaternary structures of I_3_ are similar to the ground state, the faster the acceleration of the bimolecular CO recombination process is.^36,37^ We represent the heme–heme distances and subunit rotation angles of I_3_ of WT, K30D, F97Y, and T72V in Fig. 6. For F97Y and T72V, the subunit rotation was not significantly affected, but the heme–heme contraction in F97Y and T72V was suppressed compared to WT, which is consistent with the fact that the bimolecular CO recombination rates of I^F97Y^_3_ and I^T72V^_3_ (1300 mM^−1^ s^−2^ and 310 mM^−1^ s^−2^, respectively) are faster than that of I^WT^_3_. In K30D, however, both the heme–heme contraction and subunit rotation are limited during the R–T transition, but the bimolecular CO recombination of I^K30D^_3_ is slower than that of I^WT^_3_, unlike in F97Y and T72V. Considering that the bimolecular CO recombination process requires both the structural recovery and ligand binding, the distinct correlation in K30D may indicate that the loss of the salt bridges degrades the cooperative ligand binding affinity of K30D. Conversely, it can be considered that the salt bridges in HbI may play a functional role in ligand binding as well as the structural role.

#### Structure of the monomer intermediate of the K30D mutant

Fig. S5† shows the displacement plot comparing *i* and the dimer intermediates (I^K30D^_1_, I^K30D^_2_, and I^K30D^_3_), respectively. While *i* has an R-like structure as in the K30D dimers, there are some slight differences in the movement of the CD loop, the N- and C-termini, and the F helix. The CD loop and N- and C- termini are located in the outer regions of HbI and have high flexibility in the solution. Therefore, the flexibility of these regions can lead to different structural changes between the dimer and monomer. The F helix in the monomer is exposed to the solvent, unlike in the dimer where the F helix in one subunit and the other F helix of the other subunit come in contact with each other and form the interface.

## Conclusion

In this study, we applied TRXSS to the K30D mutant of HbI to investigate the structural and functional roles of the salt bridges located at the subunit interface in the allosteric structural transition of HbI. The kinetic analysis of the TRXSS data reveals that K30D has a kinetic framework with three structurally distinct intermediates of the dimer (I_1_, I_2_, and I_3_) as in WT. But, the kinetic model of K30D involves an additional intermediate (*i*) for the monomer, of which the fraction dramatically increases compared to WT due to the weak intersubunit interaction resulting from the loss of the salt bridges. A comparative study with WT reveals that the biphasic transition from I_2_ to I_3_, which involves allosteric structural R–T transition in WT, is accelerated in K30D. The reason for the acceleration of the process was scrutinized by subsequent structural analysis. Structural analysis using the SADSs of the three dimer intermediates shows that the tertiary and quaternary structural changes are suppressed in the I^K30D^_2_-to-I^K30D^_3_ transition compared to WT, in which the I_2_^WT^-to-I^WT^_3_ transition involves R–T structural transition with dramatic tertiary and quaternary structural changes. The structural similarity between R-like I^K30D^_2_ and I^K30D^_3_ can account for the acceleration of the biphasic I^K30D^_2_-to-I^K30D^_3_ transition. The observations from the kinetic and structural analyses indicate that the loss of salt bridges not only leads to the weakening of intersubunit interaction but also interrupts the R–T allosteric structural transition of HbI. Furthermore, the correlation between the bimolecular CO recombination rates and quaternary structures of I_3_ was inspected for K30D and WT, as well as other mutants studied by TRXSS such as F97Y and T72V. Thereby, it turned out that the bimolecular CO recombination of I_3_ is abnormally decelerated in K30D, even though I_3_ of K30D has a structure more similar to the ground state than I_3_ of WT, F97Y, and T72V. Considering that the bimolecular CO recombination process requires both the structural recovery to the ground state and the binding of CO ligands, the distinct correlation observed in K30D infers that the loss of the salt bridges degrades the ligand-binding affinity of K30D. Therefore, it can be considered that the salt bridges in HbI may play a functional role in ligand binding as well as the structural role contributing to the progression of R–T allosteric structural transition. These comparisons of the structural dynamics and kinetics of K30D and WT offer insights into the role of salt bridges. If interfacial salt bridges serve only as structural constraints to increase the rigidity between two subunits, one may expect that K30D, where the salt bridges are abolished, would have a more flexible intersubunit motion than WT. The data show the opposite; K30D has a suppressed intersubunit motion, suggesting a more sophisticated role of interfacial salt bridges beyond a simple structural glue between subunits. It should be noted that the structural changes of HbI are closely related to allosteric regulation. Once a ligand is attached to the heme of one subunit, this may trigger the change of the ligand-binding affinity of the other subunit *via* allosteric regulation. This consideration shows that the interfacial salt bridges not only assist the physical connection of two subunits but also play a critical role in the global structural signal transduction of one subunit to the other subunit *via* a series of well-organized structural transitions.

## Author contributions

H. I. directed the research. H. I. designed research. S. M. performed sample preparation. T. W. K., S. L., and S. K. performed the TRXSS experiments. M. C., J. G. K., H. K., and Y. L. analyzed the data. M. C., J. G. K., and H. I. wrote the paper, and all authors discussed the experimental results.

## Conflicts of interest

The authors declare no competing financial interests.

## Supplementary Material

SC-012-D1SC01207J-s001
