# Supplementary material for: Effect of the abolition of intersubunit salt bridges on allosteric protein structural dynamics
Source: Chem Sci. 2021 May 10;12(23):8207–17. doi: 10.1039/d1sc01207j (PMC8208487; doi:10.1039/d1sc01207j)
Supplement: SC-012-D1SC01207J-s001 [file SC-012-D1SC01207J-s001.pdf]

Electronic Supplementary Material (ESI) for Chemical Science.
This journal is © The Royal Society of Chemistry 2021

Supporting information

Effect of Abolition of Intersubunit Salt Bridges on Allosteric Protein Structural Dynamics

Minseo Choi, [a,b,‡] Jong Goo Kim, [b,‡] Srinivasan Muniyappan, [b,‡] Hanui Kim,[a,b] Tae Wu Kim,[b] Yunbeom Lee,[a,b] Sang Jin Lee,[a,b] Seong Ok Kim,[b] and Hyotcherl Ihee[*,a,b]

[a]*Department of Chemistry and KI for the BioCentury, Korea Advanced Institute of Science and Technology (KAIST), Daejeon 34141, Republic of Korea*

[b]*Center for Nanomaterials and Chemical Reactions, Institute for Basic Science (IBS), Daejeon 34141, Republic of Korea*

[*]Corresponding author. Email: hyotcherl.ihee@kaist.ac.kr

[‡]These authors contributed equally to this work.

[†]Electronic Supplementary Information (ESI) available: See DOI: 10.1039/x0xx00000x

Data processing

Two-dimensional x-ray scattering patterns were azimuthally integrated to obtain one-dimensional scattering curves as a function of the magnitude of the momentum transfer vector, $q = (4\pi / \lambda)\sin(2\theta / 2)$. To convert the scattering angle (2θ) to q, the center-of-mass position of the undulator spectrum was used as the reference wavelength (λ). Since a majority of scattering signal comes from solvent pairs or bulk solvent, the laser-induced scattering intensity changes are less than a few percent of the static scattering intensity. To extract the underlying scattering signal from solute molecules, we obtained time-resolved difference x-ray solution scattering curves at the time delay of t, $\Delta S(q, t)$, shown in Fig. S6 after careful normalization. As a normalization reference, we used a q position of 2.07 Å^{-1}, which is the isosbestic point of the water scattering curves with respect to the temperature increase, so that the difference scattering intensity at this q value is zero. The scattering contribution arising from the solvent heating in the time-resolved difference x-ray solution scattering curves was removed by the following procedures. In Fig. S8, the solution scattering difference curve at 10 ms is presented as an example confirming that the difference scattering at late time delays is mainly attributed to solvent heating. The difference scattering curve is similar to the scattering curve arising from the water thermal heating, indicating that the contribution from transiently generated species is negligible. The signal where $q > 1.0$ Å^{-1} can be represented by a thermal heating signal of bulk water, which is represented by a sum of the temperature change at a constant density $((\partial S(q) / \partial T)_\rho)$ and the density change at a constant temperature $((\partial S(q) / \partial \rho)_T)$. This fitted curve, represented by a linear combination of $((\partial S(q) / \partial T)_\rho)$ and $((\partial S(q) / \partial \rho)_T)$, was subtracted from the difference curves at all time delays to remove the solvent heating effect (see Fig. S7).

General scheme for the kinetic analysis using SVD and PCA

To extract kinetics information of intermediates and their structures from $\Delta S(q, t)$, we followed the well-established procedure, which had been applied to previous TRXSS studies on WT and various mutants of HbI, consisting of kinetic analysis using singular value decomposition (SVD) and principal component analysis (PCA). First, SVD, which is a factorization method to separate the time-dependent information from the time-independent information, was performed on the $\Delta S(q, t)$ matrix for the entire time points of 100 ps - 10 ms and the q range of 0.17 - 1.0 Å^{-1}, yielding left singular vectors (lSVs), right singular vectors (rSVs) and singular values. The lSVs, rSVs and singular values contain time-independent

information, their time dependence, and their relative significance, respectively. Then, we conducted PCA, which is a method for generating theoretical difference scattering curves using time-dependent concentration changes of the intermediates expressed with a set of variable kinetic parameters, with an assumed candidate kinetic model from the results of SVD on the data matrix. Through PCA, we optimized kinetic parameters by fitting the experimental difference scattering curves with the theoretical difference scattering curves and obtained time-dependent concentration changes and species-associated difference scattering curves (SADSs) of the corresponding intermediates.

SVD Analysis

To determine the kinetic model, we need to examine the number of distinct transient species in the dynamic process of interest and their associated rate coefficients. For this purpose, we applied the singular value decomposition (SVD) analysis and the subsequent kinetic analysis to our experimental data. From the experimental scattering curves measured at various time delays, we can build $n_q \times n_t$ matrix **A**, where n_q is the number of q points in the scattering curve at a given time-delay point and n_t is the number of time-delay points. For the data in this work, n_q and n_t are 406 and 33, respectively. Then, the matrix A can be decomposed while satisfying the relationship of A = USV^T, where **U** is an $n_q \times n_t$ matrix whose columns are called left singular vectors (lSVs) and contain time-independent q spectra, **V** is an $n_t \times n_t$ matrix whose columns are called right singular vectors (rSVs) and contain time-dependent amplitude changes of the corresponding lSVs, and S is a diagonal $n_t \times n_t$ matrix whose diagonal elements are called singular values. Since the diagonal elements (i.e. singular values) of **S**, which represent the weight of lSVs in **U**, are ordered so that $s_1 \ge s_2 \ge \cdots \ge s_n \ge 0$, (both left and right) singular vectors on more left have larger contributions to the constructed experimental data. In this manner, we can extract the time-independent scattering intensity components from the lSVs, and the time evolution of their amplitudes from the rSVs, and their relative importance from the singular values. The lSVs, when combined, can give information on the scattering curves of distinct transient species, while the rSVs contain the information on the population dynamics of the transient species. Thus, the SVD analysis provides a model-independent estimation of the number of structurally distinguishable species and the dynamics of each species. As shown in Figs. 2b and S9, the singular values and autocorrelation values of the corresponding singular vectors suggest that four singular vectors are enough to represent the

experimental data of the K30D. The contribution from the fifth singular vector and beyond becomes negligible. In this regard, the first four rSVs were simultaneously fit with the sum of five exponential functions, whose time coefficients were shared, resulting in common time constants of 4.6 (± 0.7) ns, 47 (± 13) ns, 588 (± 81) ns, 616 (± 109) μs, and 5.34 (± 5.22) ms (see Fig. 2c). In summary, SVD and the global fit of rSVs show that the kinetics involves four intermediates and five time constants.

SVD with various reduced time ranges and SVD-aided pseudo PCA analysis (SAPPA) for determining the kinetic model

To establish the kinetic framework, we used two effective methods that can greatly facilitate narrowing down the kinetic models compatible with the experimental data: (i) the numbers of species from the SVD results on various reduced time ranges and (ii) SVD-aided pseudo PCA analysis (SAPPA).[1] First of all, we obtained clues about the number of intermediates associated with each of the relaxation times by performing the SVD analyses for various reduced time ranges in addition to the whole data.[1] For the SVD with a reduced time range, the upper limit close to and containing the value of each time constant obtained from the fitting of rSVs was chosen. Accordingly, four reduced time ranges (i) from 100 ps to 5.62 ns, (ii) from 100 ps to 56.2 ns, (iii) from 100 ps to 1 μs, and (iv) from 100 ps to 1 ms were used for SVD analysis. The major lSVs from the SVD analysis for reduced and the entire ranges are shown in Fig. S10. The SVD result shows that the first time range from 100 ps to 5.62 ns has two main components, indicating that the first time constant of 4.6 ns can be explained as contributing to the transition from the first intermediate (A) to the second intermediate (B). The SVD results on the second time range from 100 ps to 56.2 ns and the third range from 100 ps to 1 μs reveal that the number of main components increases to and remains at three. Adding the third intermediate (C) satisfying this condition allows for the nine kinetic frameworks shown in Fig. S14.

To further narrow down the kinetic frameworks compatible with the experimental data, we used the SAPPA method. In SAPPA, time zones that show stationary temporal behavior in the major rSVs are identified, and the experimental data at those identified stationary time zones are used as the basis to fit the experimental data at each time delay. The number of necessary time zones is the same as the number of the main components in the time range of interest, that is, three in the time range from 100 ps to 10 μs. Inspection of the first three rSVs multiplied by corresponding singular values (Fig. 2c) shows that the time zones around 100 ps, 10 ns, and 10 μs satisfy the stationary condition (that is, the rSVs do not change much with

time). Then we used the experimental difference curves corresponding to these three time zones (more specifically, the difference curves at 100 ps, 10 ns, and 10 μs) to fit the corresponding contribution at each time delay. The obtained profiles are shown in Fig. S15a. These profiles already show the general kinetic framework containing A → B → C. At this point, it is worth noting that three, the number of main components for the second time range from 100 ps to 56.2 ns, is compatible with the parallel kinetic framework containing both A → B and A → C as well as the sequential one containing A → B → C. The SAPPA kinetic profiles rule out the parallel framework. For this reason, we showed only those with the sequential one in Fig. S14 (to avoid unnecessary complexity that may confuse readers).

While this simple framework of A → B → C needs only two time constants, three time constants obtained from rSVs (4.6 ns, 47 ns, 588 ns) indicate that there should be one more kinetic process involved. Fitting these profiles using these time constants show the most natural scenario is that 4.6 ns accounts for A → B and both 47 ns and 588 ns account for B → C, meaning that a B-to-C biphasic transition (model (6) in Fig. S14). Moreover, a comparison of the SAPPA kinetic profiles (Fig. S15b) and the expected kinetic profiles of the nine candidate frameworks (Fig. S15c) immediately allows for ruling out models (1) ~ (5), (8), and (9), whereas those of models (6) and (7) are compatible with the SAPPA profiles. Among these two models, only model (6) contains the biphasic transition from the second intermediate to the third intermediate, which is one of the common features observed in WT and all other mutants (F97Y, T72V, and I114F) studied by TRXSS. In other words, if A, B, and C are assigned to I_1, I_2, and I_3, respectively, model (6) is the same as the kinetic framework of WT, F97Y, T72V, and I114F. It should be noted that the I_2-to-I_3 biphasic transition originates from the existence of both fully photolyzed and partially photolyzed forms. The fully photolyzed form converts faster than the partially photolyzed form due to the allosteric effect. One cannot rule out the possibility of model (7), but we consider this model highly unlikely because it is difficult to find any reason justifying why only the dimer of K30D shows such a drastically different kinetic framework.

The identity of one intermediate among four remains to be determined. This remaining intermediate can be either a monomer or a dimer. Nevertheless, we favor the possibility of a "monomer" intermediate for the following reasons. First, the K30D mutant has a weakened network of interaction between Asp30 and Asp89 located in the subunit interface. In WT, there are intersubunit contacts involving the E and F helices, and the Lys30 residue, which has the form of cationic ammonium with a positive charge, is located near the linker connecting the E

and F helices. As shown in the enlarged view of the interface with the two residues in Fig. 1, Lys30 in one subunit and Asp89 in the other subunit interact with each other by forming salt bridges and serve as bridges connecting the upper and lower subunits. The K30D mutation induces repulsive force between the negative charge of Asp30 introduced by the mutation and the negative charge of Asp89 in the other subunit, and consequently, the attraction between the two subunits is significantly weakened. The weakened interaction can increase the monomer proportion, and thus it is plausible that the additional intermediate, which was not observed for WT and mutants studied so far, is related to the monomer of the K30D. A study reported that the CO ligated form of WT has the dimerization constant of 2.2×10^8 M^{-1} at 25 °C,[2] which corresponds to the monomer ratio of 0.0003% in aqueous solution. By contrast, K30D has a dimerization constant of 1.2×10^3 M^{-1} at 10 °C[3] under the equivalent condition and the ratio of the monomer is 41% in aqueous solution. Considering that our TRXSS experiment was conducted at room temperature (25 °C), the monomer ratio should be even higher than that reported for 10 °C. Second, the K30D monomer can be regarded as a monomeric heme protein such as myoglobin and cytochrome c, whose photoinduced structural changes were observed in many time-resolved studies,[4-19] and thus it is plausible that the K30D monomer undergoes the structural change induced by photoexcitation. Third, the simulated difference scattering intensity of the monomer showed a magnitude that cannot be neglected compared to that of the dimer. To check if TRXSS data can be sensitive to the structural change of the monomer, we simulated the difference scattering curves by simply splitting the carboxy form and intermediates of WT HbI in half and assuming that the same structural changes occurring in the WT dimer occur in the WT monomer (Fig. S3). Surprisingly, even if the monomer is half the dimer's size, the difference scattering intensity of the monomer is similar to that of the dimer in the q range of 0.17 - 1.0 $Å^{-1}$, or even larger in certain q regions. Therefore, the kinetic analysis was performed assuming a framework that included the photoinduced structural change of the dimeric form of K30D $HbI(CO)_2$ and the monomeric form of K30D $HbI(CO)_2$.

Assignment of time constants for bimolecular CO recombination

To discuss the kinetic model containing the monomer, we consider the issue of assigning time constants of 616 μs and 5.34 ms. In previous studies for most of the heme proteins such as the WT HbI, its mutants, Hb, and Mb, the bimolecular rate of the recovery from the last intermediate (I_3 in the case of HbI) to the initial CO-liganded state was approximated as a biexponential function using two rate constants observed in rSVs.[20-22] For the same approach to be applied to the case of K30D, where two intermediates, I_3 and i, instead

of a single intermediate, undergo bimolecular CO recombination, four time constants (two per each intermediate) are required whereas only two time constants (616 μs and 5.34 ms) were identified from SVD. To identify the root of this situation, we checked the difference signal of the whole time delay and noticed that the difference curve at 10 ms still has a remaining difference signal, which means that the CO recombination is not fully completed within the time range of our experiment. If the data had been collected at time delays later than 10 ms, time constants larger than 5.34 ms would have been extracted, giving exponential time constants more than two. Considering this fact and given the possible combinations of the two time constants, the situations that best describe the data are the following two cases. In the first case, model (a) (Fig. S11a), the time constants of 616 μs and 5.34 ms are assigned to the bimolecular CO recombination of *i* that is already generated in the time resolution (< 100 ps) and 5.34 ms being the time constant reused for I_3 returning to HbI $(CO)_2$. In the second case, model (b) (Fig. S11b), on the contrary, two time constants are assigned to CO recombination of I_3 and 5.34 ms is reused to CO recombination of *i*. Both kinetic models shown in Figs. S11a and S11b are compatible with the SVD results in the reduced and whole time ranges and fully explain the TRXSS data. The two models have the same kinetic framework, but in the case of the model (a), the CO recombination rate of I_3 is slower compared to that of *i*, and in the case of the model (b), the opposite. Among these two possible kinetic models, we finally propose the kinetic model (a) for the following reasons. Models (a) and (b) give different fractions of monomers in addition to the different recombination rates from I_3 to HbI$(CO)_2$. A TRXSS study of Mb shows that its CO recombination rate (230 $mM^{-1}s^{-1}$)[15] is faster than that of WT HbI (95 $mM^{-1}s^{-1}$). This consideration renders that the monomer of K30D is likely to have faster CO recombination than the dimer. Besides, it is natural that the fraction of the monomer is higher than that of the dimer under our experimental condition, and thus we can exclude model (b) where the monomer is almost absent (~0.3%, shown in Fig. S11b).

Kinetic analysis

From the SVD analysis and global fit of rSV, the first four singular vectors of significant singular values and five time constants were obtained. We performed the kinetic analysis using these four singular vectors and time constants. New matrices for K30D, **U'**, **V'**, and **S'**, can be defined by removing non-significant components from **U**, **V**, and **S**, respectively. In other words, **U'** is an $n_q \times 4$ matrix containing only the first four left singular vectors of **U**, **S'** is a 4×4 diagonal matrix containing the first four singular values of **S**, and **V'** is an $n_q \times 4$ matrix

containing the first four right singular vectors of **V**. Here we represent the time-dependent concentrations of transiently formed intermediate species, which can be calculated from a kinetic model, by a matrix **C**. Then, the matrix **C** can be related to **V'** by using a parameter matrix **P** that satisfies **V'** = **CP**. In our analysis, **C** is an $n_q \times 4$ matrix containing the time-dependent concentrations of four intermediates of K30D HbI, that is, the dimer intermediates of I_1, I_2, I_3, and the monomer intermediate of *i*, and **P** is a 44 matrix containing coefficients for the time-dependent concentrations so that the linear combination of concentrations of the three intermediates can form the three right singular vectors in **V'**. Once **C** is specified by a kinetic model with a certain set of variable kinetic parameters such as rate coefficients, **P** and **C** can be optimized by minimizing the discrepancy between **V'** and **CP**. However, standard deviations for **V'** are not available from the experimental data, and thus we instead used the following method to optimize **P** and **C**. Since **V'** = **CP**, the following equation holds.

$$\mathbf{A}' = \mathbf{U}'\mathbf{S}'\mathbf{V}' = \mathbf{U}'\mathbf{S}'(\mathbf{CP})^{\mathrm{T}} = \mathbf{U}'\mathbf{S}'(\mathbf{P}^{\mathrm{T}}\mathbf{C}^{\mathrm{T}}) = (\mathbf{U}'\mathbf{S}'\mathbf{P}^{\mathrm{T}})\mathbf{C}^{\mathrm{T}} \quad (1)$$

where **A'** is an $n_q \times n_t$ matrix that contains the theoretical difference scattering curve $\Delta S_{theory}(q_i, t_j)$ at given *q* and *t* values. Theoretical difference scattering curves calculated by using Eqn. (1) were compared with the experimental difference scattering curves, and the matrices **P** and **C** were optimized by minimizing the discrepancy χ^2 between the theoretical and experimental difference scattering curves. From Eqn. (1), we can define a matrix **B** as **B** = **U'S'P**^{T}, that is, a linear combination of the three left singular vectors in **U'** weighted by their singular values in **S'** with their ratios determined by **P**. Then, the matrix **B**, an $n_q \times 4$ matrix, contains the four difference scattering curves directly associated with the four intermediate species of K30D HbI. As a result of the kinetics analysis, we could determine the optimum kinetic parameters (Table S1) and extract the time-independent SADSs (Fig. 3b) and time-dependent concentration changes (Fig. 3c) of the four intermediates.

Generation of the template structures for the structural analysis

Up to date, TRXSS studies on HbI have been performed with structure refinement applied with a rigid-body modeling approach using crystallographic structures as template structures.[22-25] In the case of K30D, however, this method could not be applied because crystallographic structures were not reported, unlike WT or other mutants. HbI is a homodimeric protein with two symmetric subunits consisting of 16 α-helices, 14 linkers, and two heme groups. The proportion of α-helices accounts for about 80% of the total number of

residues, and the protein is considered to be a relatively rigid protein because the α-helices are closely packed together. Also, the visible absorption spectra and circular dichroism spectra of WT and K30D HbI are nearly identical to each other for both liganded and unliganded forms, indicating high similarity in the local structure near the heme pocket in WT and K30D HbI.[3] We, therefore, assumed that the backbone of WT and K30D would be quite similar to each other and generated hypothetical crystallographic structures of K30D by incorporating single amino acid residue replacements from the crystallographic structure formed at 5 ns after the photolysis of carbonyl ligand (PDB ID: 2GRZ) and the crystallographic structure of the carboxy form of WT HbI (PDB ID: 3SDH). The resulting structures were used as template structures in the structure refinement for K30D. The original crystallographic structures were modified using PyMol software by replacing Lys30 residues of two subunits of the HbI with Asp30 residues.

Structure refinement

The structure refinement using SADSs of all intermediates of the K30D was performed. The positions and orientations of the rigid bodies were randomly generated based on a Monte Carlo simulation algorithm and refined to minimize the discrepancy between the theoretical difference scattering curve calculated from the refined structure and the SADSs of the dimer and monomer intermediates. For each intermediate, the refinement process was repeated for 360 different initial structures whose rigid bodies were randomly displaced from the template. We selected 195, 167, 200, and 196 candidate structures for the I_1, I_2, I_3 and i, respectively, which exhibited χ^2 values (a quantified value of the discrepancy between the experimental and theoretical difference scattering curves) below a certain threshold. The theoretical difference scattering curves for the refined candidate structures are shown in Fig. S4.

Supporting information table and figures

Table S1 Kinetic parameters obtained from the kinetic analysis of TRXSS data of K30D.

**Parameters**	**Fit values**
Time constant for I_1-to-I_2 transition	4.6 (± 0.7) ns
Fast time constant for I_2-to-I_3 transition	47 (± 13) ns
Slower time constant for I_2-to-I_3 transition	558 (± 81) ns
Rate constant for bimolecular CO recombination of dimer (I_3)	36.1 (± 3.31) $mM^{-1}s^{-1}$
Rate constant for bimolecular CO recombination of monomer (*i*)	309 (± 8.57) $mM^{-1}s^{-1}$
Fraction of dimer	19 (± 0.41) %
Fraction of fully photolyzed forms	38 (± 0.006) %

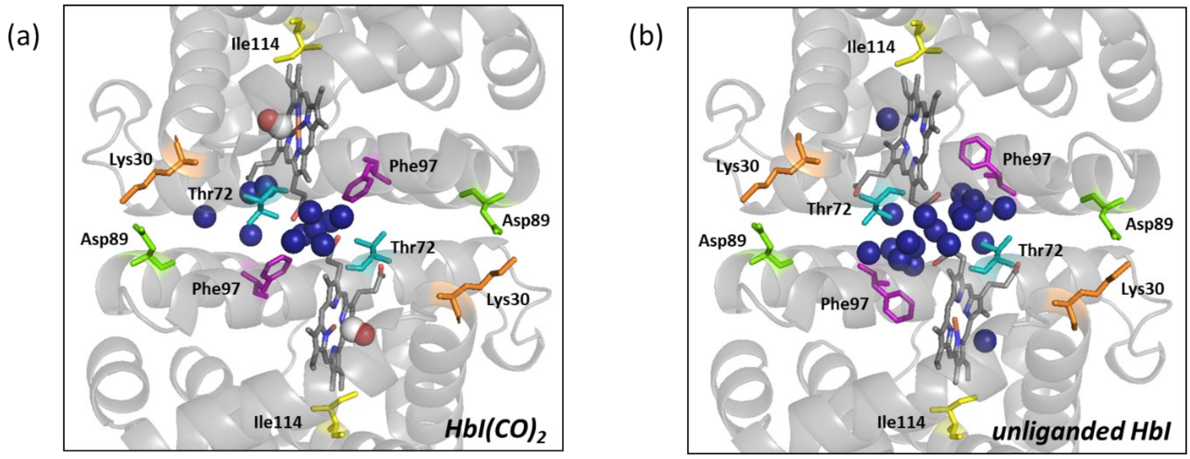

Fig. S1 (a) Enlarged view of the crystal structure of WT HbI$(CO)_2$ (PDB ID: 3SDH) and (b) unliganded WT HbI (PDB ID: 4SDH). Eleven and seventeen interfacial water molecules for HbI$(CO)_2$ and unliganded HbI, respectively, are shown with blue spheres. Heme-bounded CO molecules are shown with connected red and white spheres. The residues regulating the cooperative ligand binding and allosteric structural transition of HbI are indicated by sticks. Phe97, Thr72, Ile114, Lys30, and Asp89 are represented in purple, teal, yellow, orange, and light green colors, respectively.

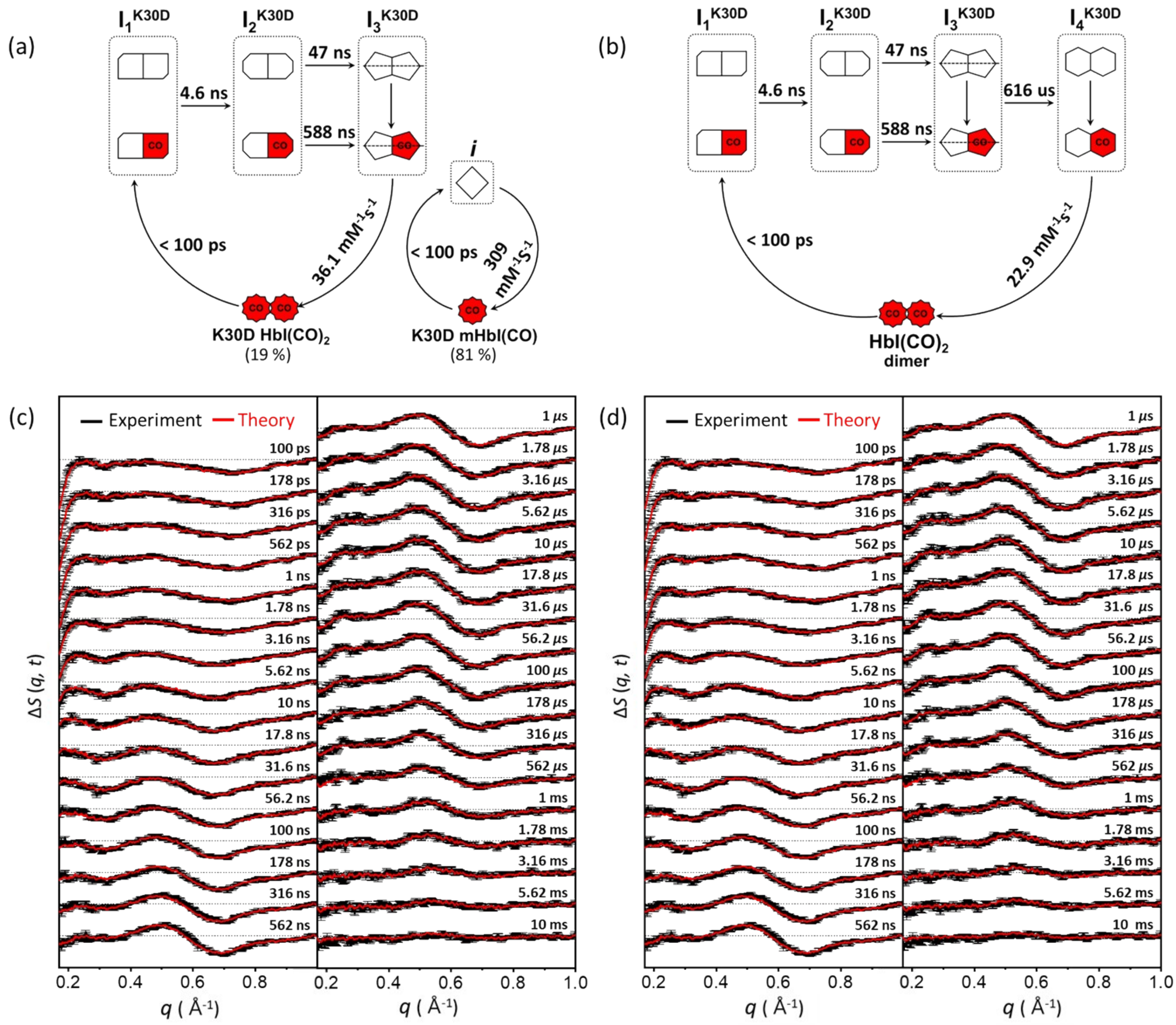

Fig. S2 (a, b) Kinetic models for the two scenarios where the fourth species are assumed to be (a) the monomer intermediate, *i*, and (b) the dimer intermediate, I_4^{K30D}. (c, d) Time-resolved x-ray solution scattering curves, $\Delta S(q, t)$, measured for a solution sample of K30D (black curves) are compared with theoretical curves (red curves) generated by linear combinations of left singular vectors (lSVs) based on the kinetic models where the fourth species is assumed to be (c) the monomer intermediate, *i,* and (d) the dimer intermediate, I_4^{K30D}. The calculated curves from both scenarios give equally satisfactory agreement with the experimental data.

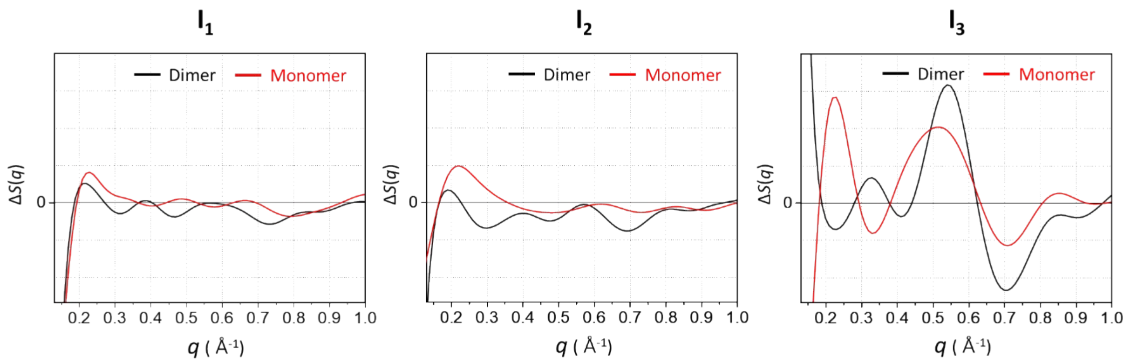

Fig. S3 Simulated difference scattering curves for the structural transition of the monomer and dimer of WT HbI for all three intermediates (I_1, I_2, and I_3). To simulate the structural transition of the monomer, both the crystallographic structure of the liganded form (PDB ID: 3SDH) and the intermediates of WT HbI were simply split into two halves and the corresponding scattering curve was calculated for the generated monomers of the liganded form and intermediates with the assumption that the same structural changes occurring in the WT dimer occur in the WT monomer. Difference scattering curves of the dimer are shown in black and the monomer difference scattering curves of the monomer are shown in red.

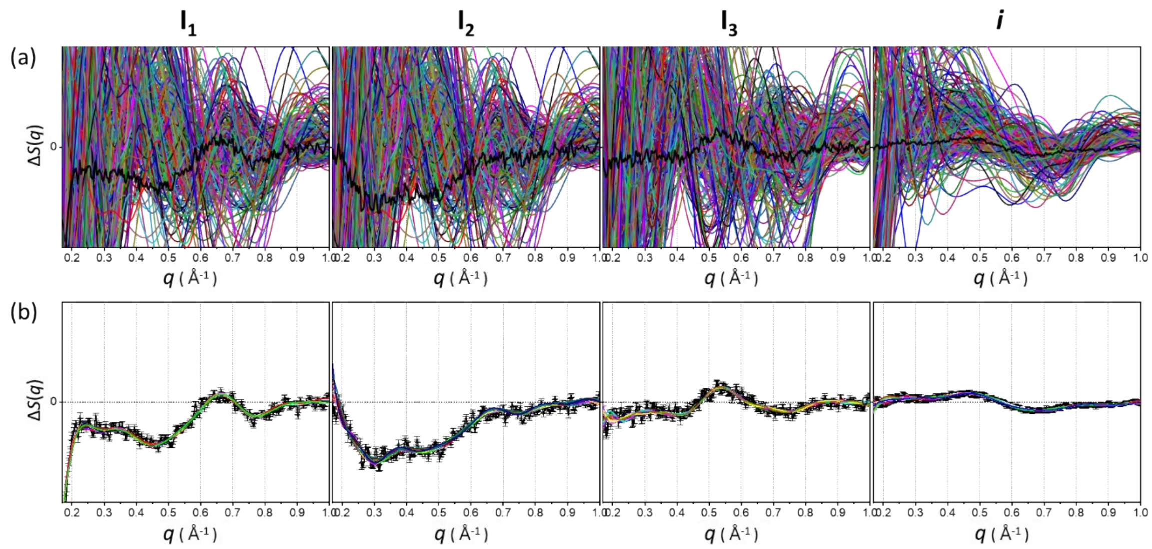

Fig. S4 (a) Comparison of the experimental SADSs of all intermediates I_1, I_2, I_3, *i* (black curves) with the theoretical scattering curves of the 360 randomly generated initial structures (color lines). (b) Comparison of the experimental SADSs of all intermediates I_1, I_2, I_3, *i* (black lines) with the experimental standard deviation and the theoretical scattering curves (color lines) from the best fit structures.

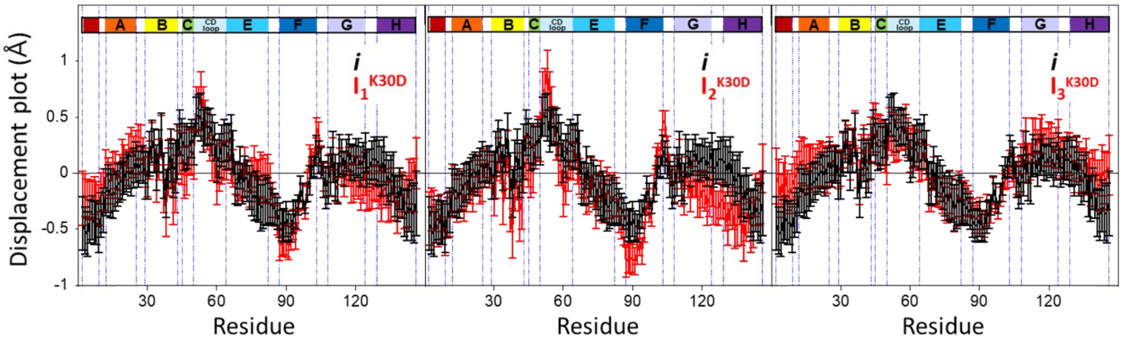

Fig. S5 The averaged displacement plots of the candidate structures for the monomer intermediate *i* (black), and the dimer intermediates (red) for I_1^{K30D} (left), I_2^{K30D} (middle), and I_3^{K30D} (right) were calculated with respect to the half of the mutated liganded crystal structure of WT (mutated from 3SDH). Error bars represent standard deviation values among various candidate structures of each intermediate.

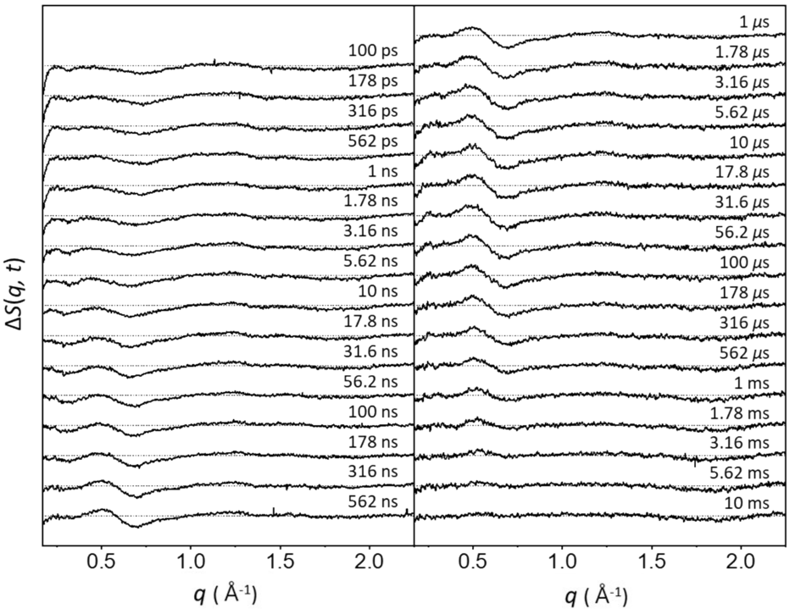

Fig. S6 Time-resolved difference x-ray solution scattering curves of K30D. We used q position of 2.07 $Å^{-1}$ as a normalization reference so that the difference scattering intensity at this q point is zero.

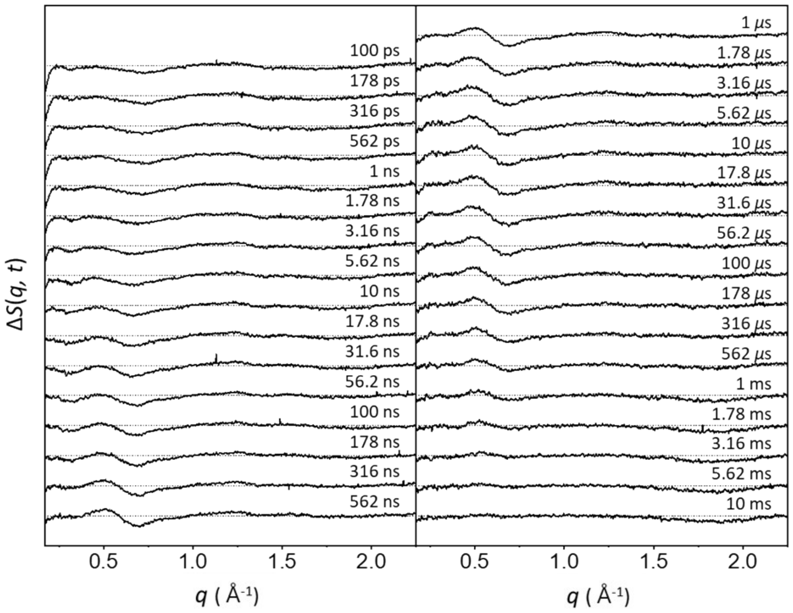

Fig. S7 Heat-free, time-resolved difference x-ray solution scattering data of K30D. The thermal heating signal of bulk water shown in Fig. S8 was subtracted from the original difference curves shown in Fig. S6.

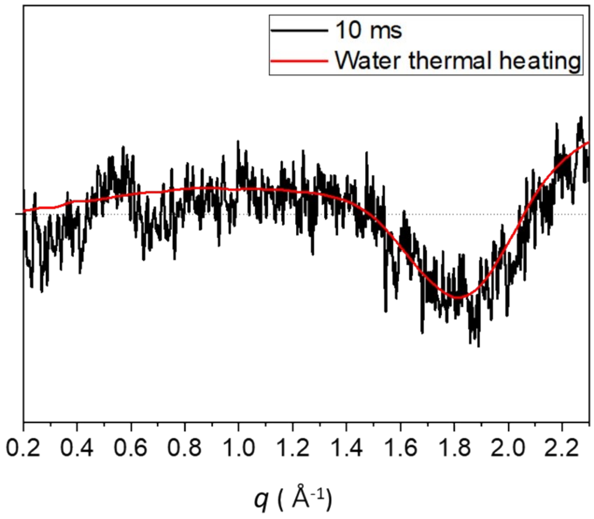

Fig. S8 The difference solution scattering curve at 10 ms (black) time delay. At this time delay, the difference scattering curve is similar to the scattering intensity change upon a temperature increase of the water solvent (red), indicating that the contribution from transiently generated species is negligible. More specifically, the signal in the region where q > 1.0 Å^{-1} can be represented by a heating signal of bulk water, which can be represented by a sum of the temperature change at constant density ($(\partial S(q)/\partial T)_\rho$) and the density change at constant temperature ($(\partial S(q)/\partial \rho)_T$). In other words, the difference signals at late time delays are mainly attributed to solvent heating.

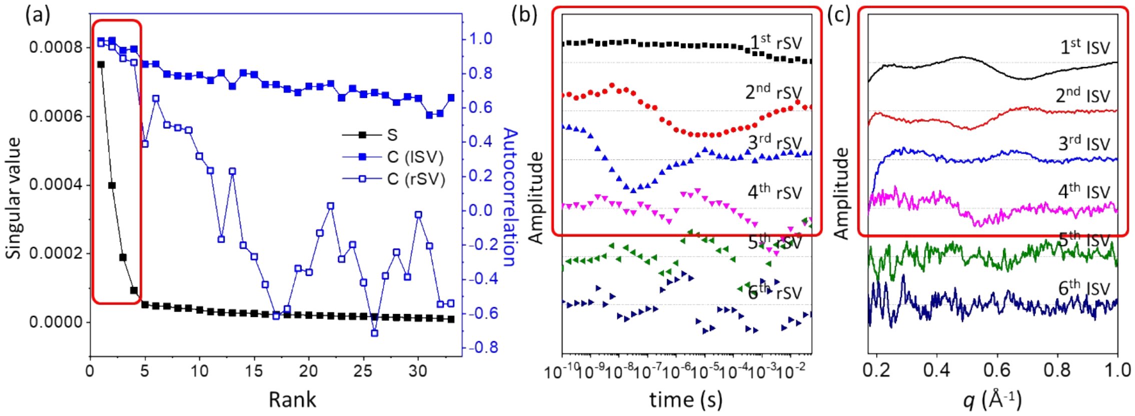

Fig. S9 (a) Singular values (solid black square), autocorrelations of left singular vectors, lSV, (solid blue square) and right singular vectors, rSVs, (blue open square) were obtained from TRXSS data. The first four (both left and right) singular vectors were selected for our kinetic analysis. (b) The first six rSVs are shown. (c) The first six rSVs are shown.

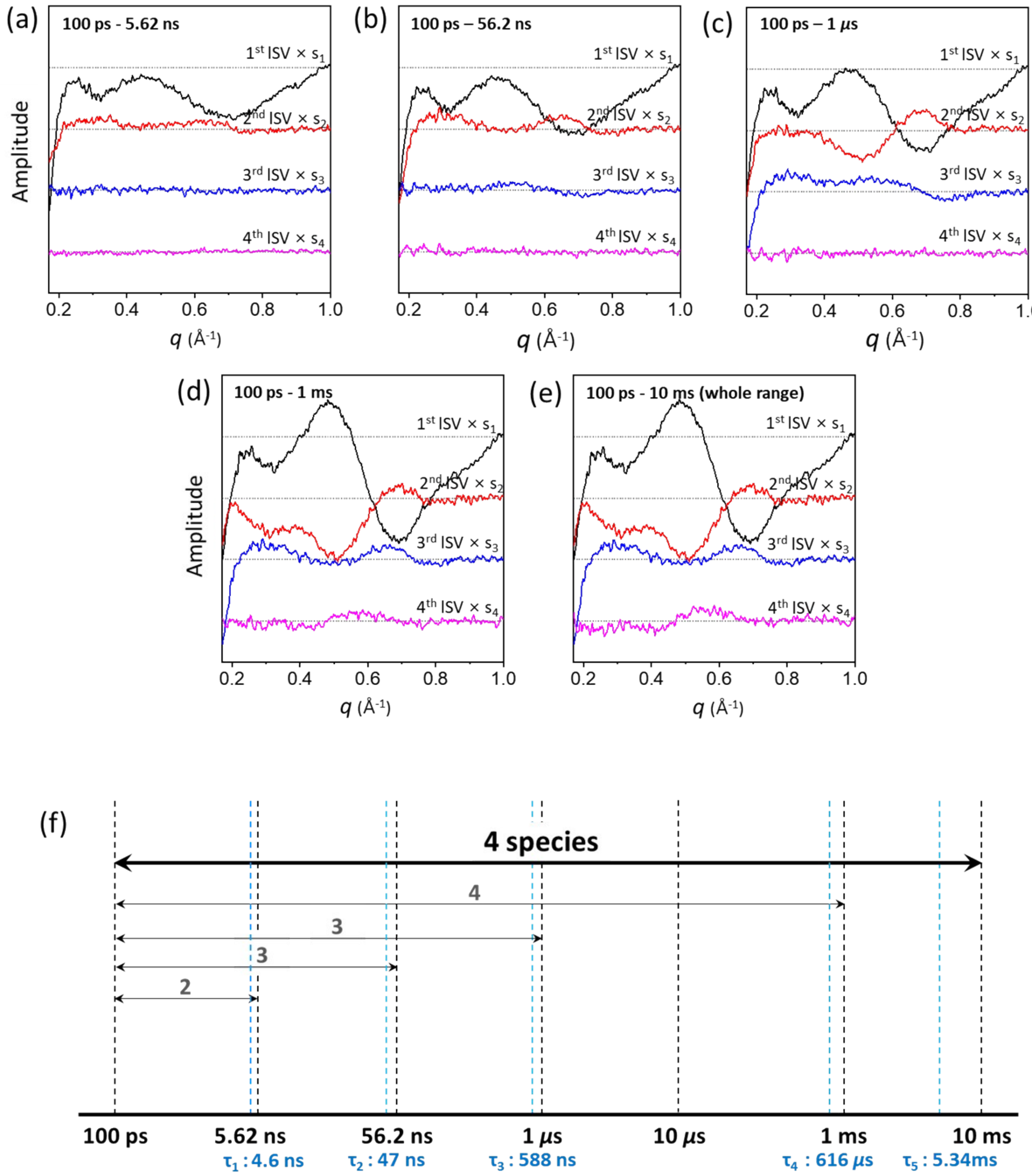

Fig. S10 The result of SVD analysis in six different time ranges. The first four lSVs multiplied by singular values in the reduced time ranges of (a) 100 ps - 5.62 ns, (b) 100 ps - 56.2 ns, (c) 100 ps - 1 μs, (d) 100 ps - 1 ms, and (e) entire time range. As shown in Fig. (a), for example, two singular components of significant amplitudes were identified in this reduced time range of 100 ps - 5.62 ns. Various reduced time ranges, whose upper limit is close to and containing the value of each time constant, are shown in (f).

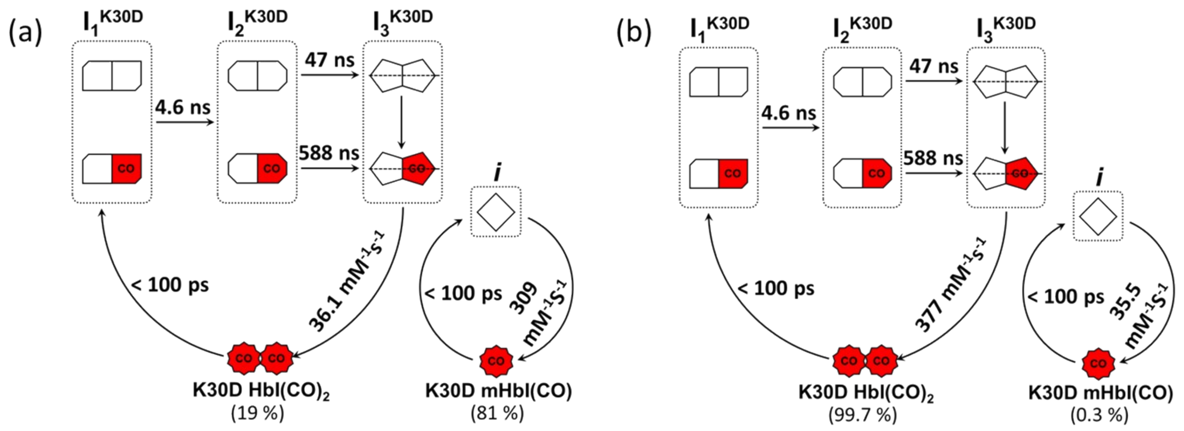

Fig. S11 Kinetic models for K30D containing a monomer intermediate, *i*, compatible with the SVD results in various time ranges. The red (with "CO") and white symbols represent liganded and photolyzed subunits, respectively. To indicate the four structurally distinguishable intermediates with different tertiary structures, the subunit of each intermediate is presented with a different shape. In the kinetic model shown in (a), the bimolecular CO recombination of the monomer is faster than that of the dimer. In contrast, in the kinetic model shown in (b), the bimolecular CO recombination of the monomer is slower than that of the dimer.

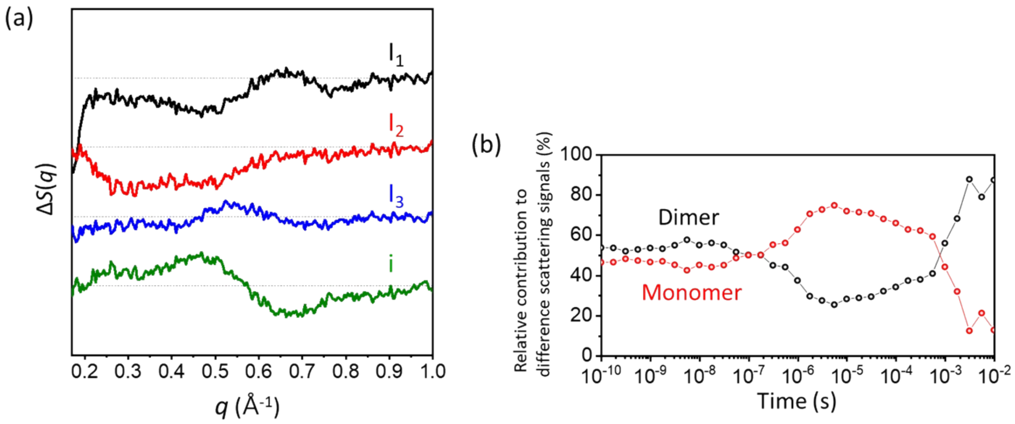

Fig. S12 (a) SADSs multiplied by the maximum concentrations of the four species (0.70 mM for I_1, 0.64 mM for I_2, 0.72 mM for I_3, and 2.91 mM for *i*). This figure confirms that the contribution of dimers is comparable to that of monomers. More importantly, the magnitudes of SADSs are substantially larger than the noise level, indicating that the signal-to-noise ratio of the current data is enough to provide information for both dimers and monomers. (b) The relative contributions of dimers and monomers to the difference scattering signals as a function of time.

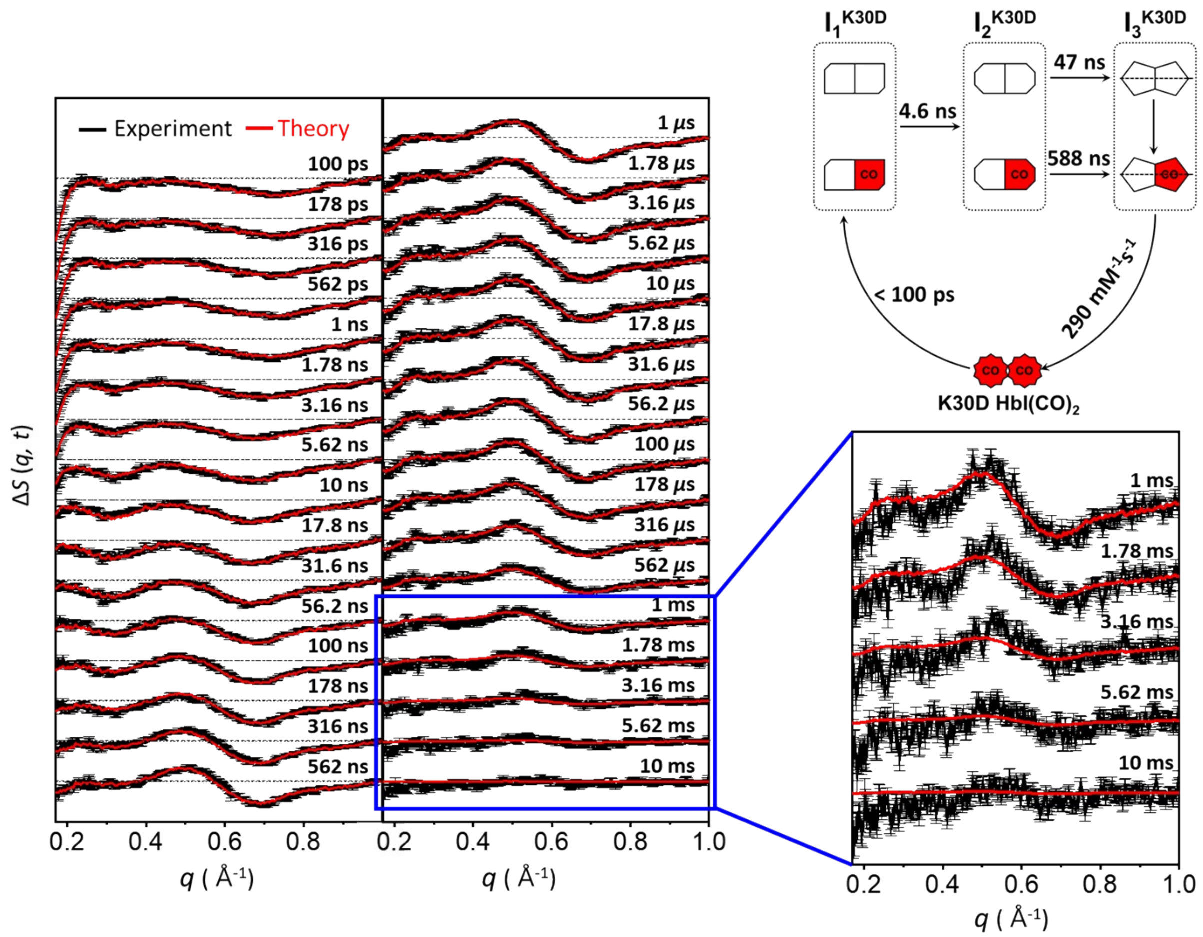

Fig. S13 Time-resolved x-ray solution scattering curves, ΔS(*q*, *t*), measured for a solution sample of K30D (black curves) are compared with theoretical curves (red curves) generated by linear combinations of only three lSVs instead of four. based on the kinetic model with only three dimer intermediates (I_1, I_2, and I_3) without the monomer intermediate (*i*). Note that the fit quality is significantly worse than those including the fourth intermediate in Fig. S2. The chi-square value increases by 21%.

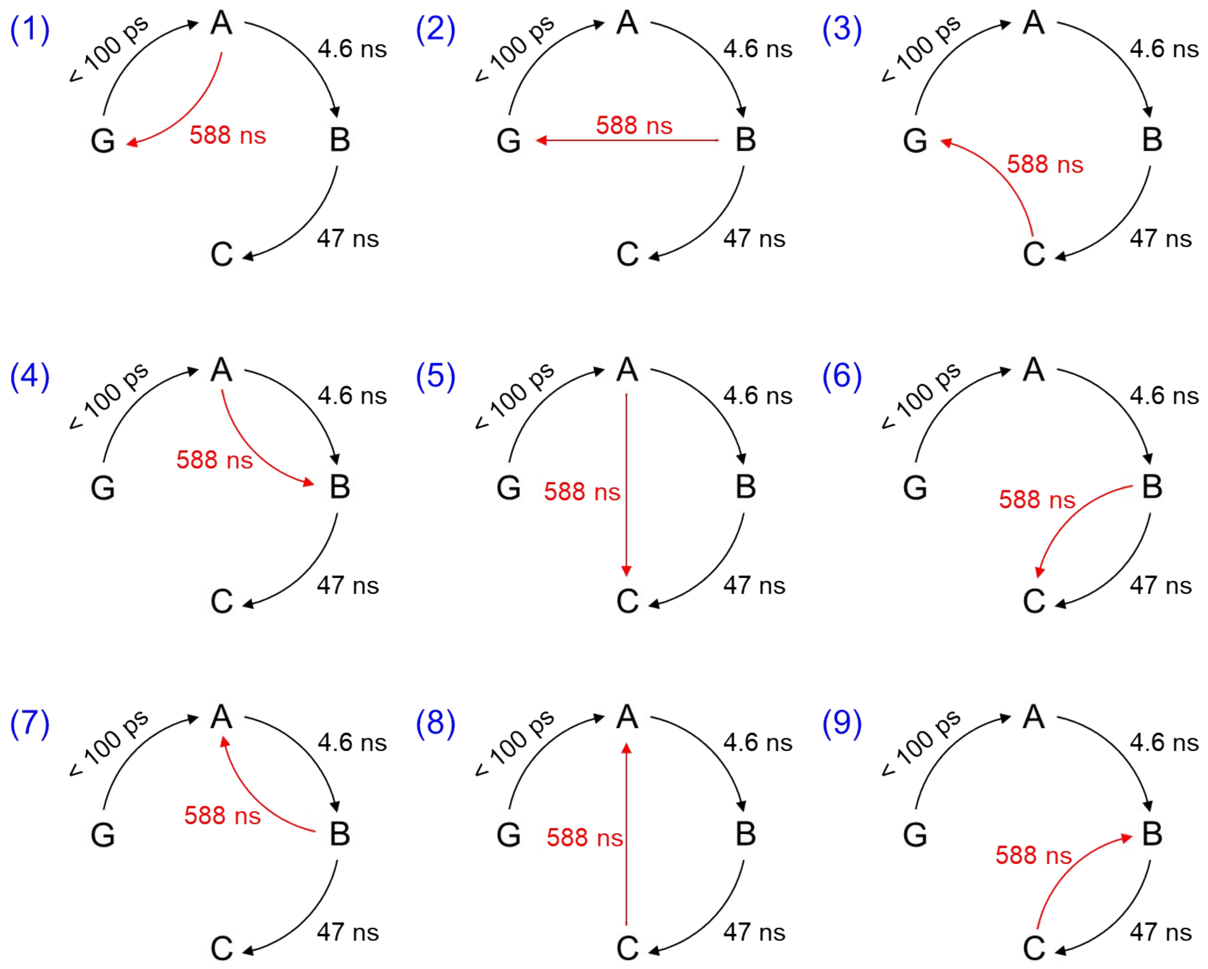

Fig. S14 All possible candidate kinetic models for the time range from 100 ps to 10 μs, containing the sequential kinetic framework of G → A → B → C indicated by black arrows, where G, A, B, and C refer to the ground state, the first intermediate, the second intermediate, and the third intermediate, respectively. In all kinetic models, time constants of 4.6 ns and 47 ns are commonly assigned to A → B and B → C transitions, respectively, to be compatible with the results of SVD analyses in the reduced time ranges shown in Fig. S10. The kinetic models (1) ~ (9) are distinguished from each other in terms of the assignment of the time constant of 588 ns. In each kinetic model, the assignment of 588 ns is indicated by a red arrow.

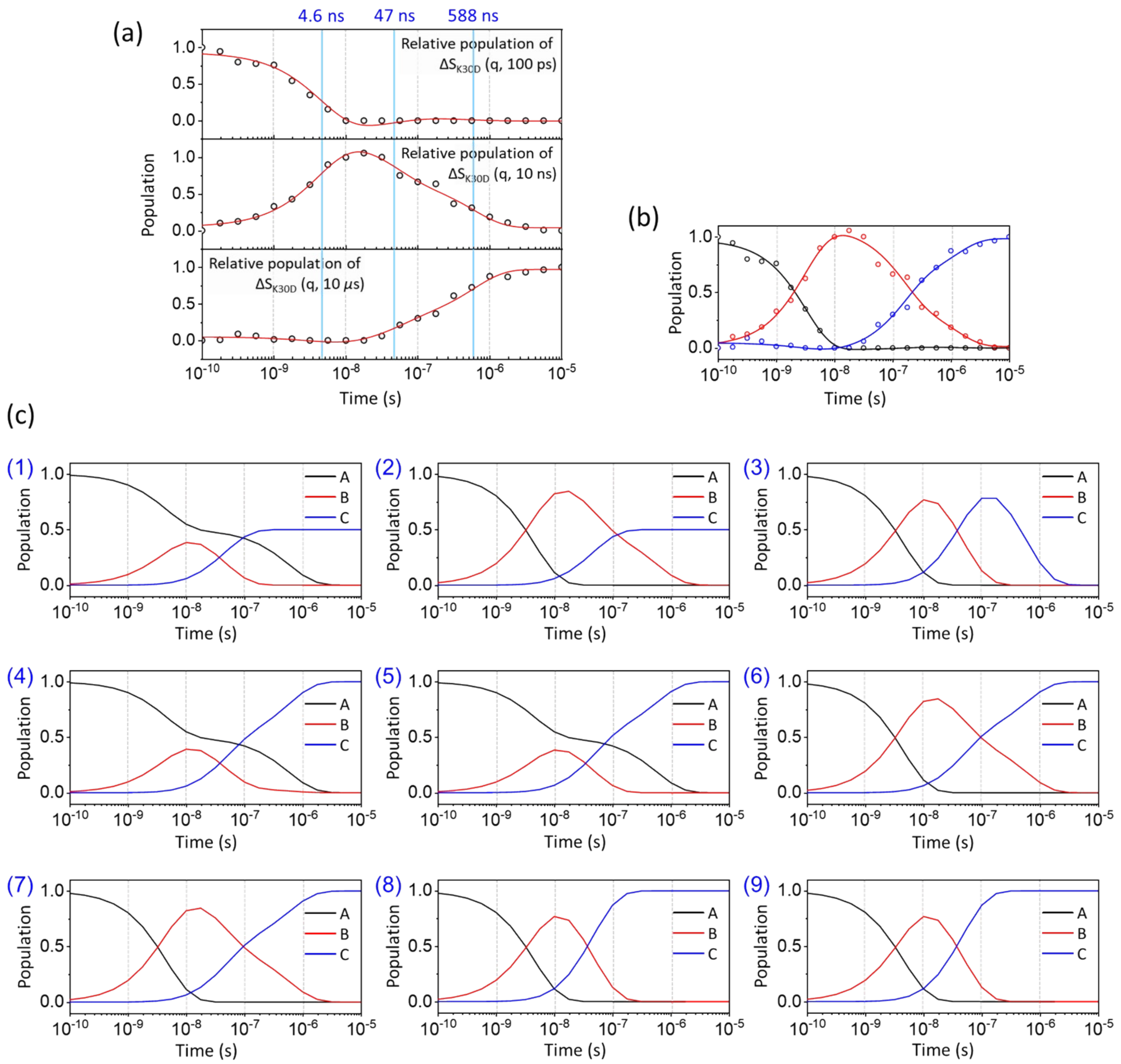

Fig. S15 (a) The SAPPA kinetic profiles obtained from SAPPA using the experimental difference curves at 100 ps, 10 ns, and 10 μs as the basis spectra to fit the relative contributions of the three basis spectra as a function of time. The relative populations indicated in empty circles are obtained from SAPPA, and the red curves are from fitting the three SAPPA kinetic profiles with the common time constants fixed at those obtained from the fit of rSVs (4.6 ns, 47 ns, 588 ns). (b) The SAPPA kinetic profiles of (a) shown in a different style to facilitate easy comparison with the kinetic profiles from candidate kinetic models of (c). The profiles of the first, second, and third basis components are shown in black, red, and blue, respectively. (c) The simulated kinetic profiles of three intermediates for the nine candidate kinetic models shown in Fig. S14. Comparison of the kinetic profiles in (c) and the SAPPA kinetic profiles in (b) show that only models (6) and (7) are compatible with the experimental data, ruling out the other models.

REFERENCES

1. K. Y. Oang, C. Yang, S. Muniyappan, J. Kim and H. Ihee, *Struct. Dyn.*, 2017, **4**, 044013.
2. W. E. Royer, Jr., R. A. Fox, F. R. Smith, D. Zhu and E. H. Braswell, *J. Biol. Chem.*, 1997, **272**, 5689-5694.
3. P. Ceci, L. Giangiacomo, A. Boffi and E. Chiancone, *J. Biol. Chem.*, 2002, **277**, 6929-6933.
4. V. Srajer, T. Teng, T. Ursby, C. Pradervand, Z. Ren, S. Adachi, W. Schildkamp, D. Bourgeois, M. Wulff and K. Moffat, *Science*, 1996, **274**, 1726-1729.
5. E. Chen, M. J. Wood, A. L. Fink and D. S. Kliger, *Biochemistry*, 1998, **37**, 5589-5598.
6. V. Srajer, Z. Ren, T. Y. Teng, M. Schmidt, T. Ursby, D. Bourgeois, C. Pradervand, W. Schildkamp, M. Wulff and K. Moffat, *Biochemistry*, 2001, **40**, 13802-13815.
7. F. Schotte, M. Lim, T. A. Jackson, A. V. Smirnov, J. Soman, J. S. Olson, G. N. Phillips, Jr., M. Wulff and P. A. Anfinrud, *Science*, 2003, **300**, 1944-1947.
8. M. Schmidt, K. Nienhaus, R. Pahl, A. Krasselt, S. Anderson, F. Parak, G. U. Nienhaus and V. Srajer, *Proc. Natl. Acad. Sci. U. S. A.*, 2005, **102**, 11704-11709.
9. R. t. Aranda, E. J. Levin, F. Schotte, P. A. Anfinrud and G. N. Phillips, Jr., *Acta Crystallogr., Sect. D: Biol. Crystallogr.* , 2006, **62**, 776-783.
10. M. Cammarata, M. Levantino, F. Schotte, P. A. Anfinrud, F. Ewald, J. Choi, A. Cupane, M. Wulff and H. Ihee, *Nat. Methods*, 2008, **5**, 881-886.
11. S. Ahn, K. H. Kim, Y. Kim, J. Kim and H. Ihee, *J. Phys. Chem. B*, 2009, **113**, 13131-13133.
12. H. S. Cho, N. Dashdorj, F. Schotte, T. Graber, R. Henning and P. Anfinrud, *Proc. Natl. Acad. Sci. U. S. A.*, 2010, **107**, 7281-7286.
13. S. Westenhoff, E. Nazarenko, E. Malmerberg, J. Davidsson, G. Katona and R. Neutze, *Acta Crystallogr., Sect. A: Found. Crystallogr.*, 2010, **66**, 207-219.
14. K. H. Kim, K. Y. Oang, J. Kim, J. H. Lee, Y. Kim and H. Ihee, *Chem. Commun.*, 2011, **47**, 289-291.
15. K. Y. Oang, J. G. Kim, C. Yang, T. W. Kim, Y. Kim, K. H. Kim, J. Kim and H. Ihee, *J. Phys. Chem. Lett.*, 2014, **5**, 804-808.
16. K. Y. Oang, K. H. Kim, J. Jo, Y. Kim, J. G. Kim, T. W. Kim, S. Jun, J. Kim and H. Ihee, *Chem. Phys.*, 2014, **422**, 137-142.
17. J. G. Kim, T. W. Kim, J. Kim and H. Ihee, *Acc. Chem. Res.*, 2015, **48**, 2200-2208.
18. A. Shimada, M. Kubo, S. Baba, K. Yamashita, K. Hirata, G. Ueno, T. Nomura, T. Kimura, K. Shinzawa-Itoh, J. Baba, K. Hatano, Y. Eto, A. Miyamoto, H. Murakami, T.

Kumasaka, S. Owada, K. Tono, M. Yabashi, Y. Yamaguchi, S. Yanagisawa, M. Sakaguchi, T. Ogura, R. Komiya, J. Yan, E. Yamashita, M. Yamamoto, H. Ago, S. Yoshikawa and T. Tsukihara, *Sci. Adv.*, 2017, **3**, e1603042.

19. T. W. Kim, S. J. Lee, J. Jo, J. G. Kim, H. Ki, C. W. Kim, K. H. Cho, J. Choi, J. H. Lee, M. Wulff, Y. M. Rhee and H. Ihee, *Proc. Natl. Acad. Sci. U. S. A.*, 2020, **117**, 14996-15005.
20. A. Ansari, C. M. Jones, E. R. Henry, J. Hofrichter and W. A. Eaton, *Biochemistry*, 1994, **33**, 5128-5145.
21. G. Balakrishnan, M. A. Case, A. Pevsner, X. Zhao, C. Tengroth, G. L. McLendon and T. G. Spiro, *J. Mol. Biol.*, 2004, **340**, 843-856.
22. K. H. Kim, S. Muniyappan, K. Y. Oang, J. G. Kim, S. Nozawa, T. Sato, S. Y. Koshihara, R. Henning, I. Kosheleva, H. Ki, Y. Kim, T. W. Kim, J. Kim, S. Adachi and H. Ihee, *J. Am. Chem. Soc.*, 2012, **134**, 7001-7008.
23. J. G. Kim, S. Muniyappan, K. Y. Oang, T. W. Kim, C. Yang, K. H. Kim, J. Kim and H. Ihee, *Struct. Dyn.*, 2016, **3**, 023610.
24. C. Yang, M. Choi, J. Kim, H. Kim, S. Muniyappan, S. Nozawa, S.-i. Adachi, R. Henning, I. Kosheleva and H. Ihee, *Int. J. Mol. Sci.*, 2018, **19**, 3633.
25. H. Kim, J. G. Kim, S. Muniyappan, T. W. Kim, S. J. Lee and H. Ihee, *J. Phys. Chem. B*, 2020, **124**, 1550-1556.
